# The Vimentin-Targeting Drug ALD-R491 Partially Reverts the Epithelial-to-Mesenchymal Transition and Vimentin Interactome of Lung Cancer Cells

**DOI:** 10.3390/cancers17010081

**Published:** 2024-12-30

**Authors:** Marieke Rosier, Anja Krstulović, Hyejeong Rosemary Kim, Nihardeep Kaur, Erhumuoghene Mary Enakireru, Deebie Symmes, Katalin Dobra, Ruihuan Chen, Caroline A. Evans, Annica K. B. Gad

**Affiliations:** 1 Department of Oncology-Pathology, Karolinska Institutet, 171 64 Solna, Sweden; rosiermarieke@gmail.com (M.R.); anjakrstulovic@gmail.com (A.K.); nihardeep.kaur@stud.ki.se (N.K.); enakireruerhumuoghene@gmail.com (E.M.E.); katalin.dobra@ki.se (K.D.); 2 Department of Oncology and Metabolism, The Medical School, University of Sheffield, Sheffield S10 2RX, UK; h.r.kim@sheffield.ac.uk; 3 Aluda Pharmaceuticals, Inc., Menlo Park, CA 94025, USA; deebie@aludapharm.com (D.S.); ruihuan@aludapharm.com (R.C.); 4 School of Materials, Chemical and Biological Engineering, University of Sheffield, Sheffield S10 2TN, UK; caroline.evans@sheffield.ac.uk,

**Keywords:** vimentin intermediate filaments, vimentin interactome, extracellular matrix, epithelial-to-mesenchymal transition, TGF-β1, cell migration, carcinoma, lung cancer

## Abstract

**Background:** The epithelial-to-mesenchymal transition (EMT) is a common feature in early cancer invasion. Increased vimentin is a canonical marker of the EMT; however, the role of vimentin in EMT remains unknown. **Methods:** To clarify this, we induced EMT in lung cancer cells with TGF-β1, followed by treatment with the vimentin-targeting drug ALD-R491, live-cell imaging, and quantitative proteomics. **Results:** We identified 838 proteins in the intermediate filament fraction of cells. TGF-β1 treatment increased the proportion of vimentin in this fraction and the levels of 24 proteins. Variants of fibronectin showed the most pronounced increase (137-fold), followed by regulators of the cytoskeleton, cell motility, and division, such as the mRNA-splicing protein SON. TGF-β1 increased cell spreading and cell migration speed, and changed a positive correlation between cell migration speed and persistence to negative. ALD-R491 reversed these mesenchymal phenotypes to epithelial and the binding of RNA-binding proteins, including SON. **Conclusions:** These findings present many new interactors of intermediate filaments, describe how EMT and vimentin filament dynamics influence the intermediate filament interactome, and present ALD-R491 as a possible EMT-inhibitor. The observations support the hypothesis that the dynamic turnover of vimentin filaments and their interacting proteins govern mesenchymal cell migration, EMT, cell invasion, and cancer metastasis.

## Simple Summary

To clarify the role of the intermediate filament protein vimentin in the epithelial-to-mesenchymal transition (EMT), we induced EMT in lung cancer cells with TGF-β1, followed by treatment with the drug ALD-R491, which targets vimentin. Our findings present many new interactors of intermediate filaments, describe how vimentin filament dynamics influence the filament interactome and EMT, and present ALD-R491 as a possible EMT-inhibitor.

## 1. Introduction

The epithelial-to-mesenchymal transition (EMT) is the process in which stationary epithelial cells become elongated, motile, bind to the extracellular matrix (ECM), and express mesenchymal cell markers. This process is instrumental for embryonic development and tissue regeneration, as well as for pathological conditions, such as during the initial stages of cancer invasion and metastasis. An increased level of the intermediate filament protein vimentin is a molecular marker for EMT, along with expression of fibronectin, and the switch from E- to N-cadherin expression and significant changes in cell shape and motility [1,2].

Vimentin is a protein closely linked to increased cell motility, as it is required for persistent cell migration, wound healing, and tissue regeneration [3,4,5]. Vimentin is further required for a change from a round epithelioid to an elongated cell shape, which are morphological changes that occur during EMT [6]. Vimentin is virtually absent in well- differentiated lepidic, papillary, and acinar lung cancer subtypes. In contrast, it is overexpressed in the solid subtype and in pulmonary sarcomatoid carcinoma, lung cancer subtypes that are associated with poor prognosis [7,8]. This makes vimentin a useful diagnostic marker in differentiating between the different subtypes of lung cancer. Vimentin expression is also required for the development and metastasis of lung cancer cells in mice [9,10]. Increased levels of vimentin and EMT are also further linked to resistance to treatment with tyrosine kinase inhibitors in lung adenocarcinoma [11]. In fact, EMT constitutes a major resistance mechanism and hindrance for an effective lung cancer treatment. The roles of EMT in metastasis as well as in therapy-resistance have the potential to guide future clinical decision-making processes in the personalised medicine of lung cancer.

Vimentin is known to control cell motility by various pathways. In addition to the regulation of the mechanical and physical properties of cells, vimentin enhances the function of microtubules in directed migration, and acts as a protein scaffold, which can allow protein production and interaction at the right subcellular site at the right time [12,13,14,15,16]. The observation that vimentin filaments are required to produce collagen I, the most abundant ECM protein in the body, via stabilization of collagen mRNA production, highlights the possibility that vimentin can control cell motility and EMT via increased collagen production at specific sites [17]. We have previously found that vimentin polymerisation into filaments occurs at the base of mature focal adhesions, but not of nascent cell adhesions to the ECM, and described the role of vimentin dynamics in force-transduction over focal adhesions [18,19]. Most research on vimentin is based on overexpression or knock down studies; however, vimentin is constantly exchanged between the mature vimentin filaments and the soluble pool of vimentin [5,20]. How the dynamic properties of vimentin filaments influence EMT, or the binding of proteins, is not known. We have previously synthesised a novel drug, ALD-R491 (R491), which specifically binds to vimentin without changing the total levels of vimentin; however, it reduces the dynamic exchange, increases the stability of the vimentin filaments, increases cellular contractile force, and reduces cell migration [19,21,22]. We therefore hypothesise that vimentin filament dynamics is required for EMT in lung cancer cells, via the regulation of vimentin-interacting proteins.

Our data suggest that, during the TGF-β1-induced EMT of epithelial lung cancer cells, vimentin filament dynamics is required to gain EMT phenotypes such as increased cell spreading, increased cell migration speed, and a negative correlation between cell migration speed and directionality. We further present 838 vimentin and intermediate filament-binding proteins, of which many have been previously unknown to interact with intermediate filaments. The data suggests that filament dynamics can be essential for vimentin filaments to bind to proteins previously known to regulate cancer. We also present ALD-R491 as a potential novel drug against EMT and lung cancer metastasis, and describe the changes of the vimentin interactome that accompany TGF-β1-induced EMT, with the most significant change in components of the extracellular matrix. In summary, these findings provide novel avenues for the investigation of the regulation of cell motility, EMT, and treatment of cancer metastasis.

## 2. Materials and Methods

### 2.1. Cell Culture and Treatments

A549 cell lines were purchased from ATCC (USA), cultured in Dulbecco’s modified Eagle’s medium (31330038; DMEM, Gibco, New York, NY, USA) and supplemented with 10% Fetal Bovine Serum (Biowest, Nuaillé, France) and 100 U/mL penicillin and 100 microg/mL streptomycin (Sigma-Aldrich, Burlington, MA, USA). Cells were split weekly. The TGF-β1 was dissolved in 4 nM HCl (Sigma-Aldrich, Burlington, MA, USA), and equal final concentration of HCl was used as control. ALD-R491 was synthesised and provided by Aluda Pharmaceuticals (Menlo Park, CA, USA). Vimentin is the only protein found to bind to ALD-R491 [19]. ALD-R491 was dissolved in DMSO (Thermo Fisher Scientific, Waltham, MA, USA) and used at a final concentration of 5 μM, and an equal concentration of DMSO was used as a control.

### 2.2. Antibodies and Cell Dyes

The antibodies used were as follows: anti-vimentin (V6389; Sigma Aldrich, Burlington, MA, USA), pan-cytokeratin (ab8068), anti-FN1 (ab2413) (Abcam, Cambridge, UK), anti-N-cadherin (clone 3B9, 33-3900, Invitrogen, Waltham, MA, USA), anti-GAPDH (A1978; Sigma Aldrich, Burlington, MA, USA), and Horseradish-peroxide-conjugated goat anti-mouse secondary antibodies (GtxMu-004-DHRPX; ImmunoReagents, Raleigh, NC, USA). To stain for nuclei, we used 0.05 μg/mL Hoechst and phalloidin for F-actin (both from Sigma-Aldrich, Burlington, MA, USA).

### 2.3. Western Blot

The cytoskeletal isolation fractions were separated using 4%–15% precast polyacrylamide gels (4568084; Bio-Rad, Hercules, CA, USA), with the Precision Plus Protein Dual Color Standards protein size marker (1610374, Bio-Rad, CA, USA). The gels were then transferred onto nitrocellulose membranes (1704271; Bio-Rad, Hercules, CA, USA) using a transfer system (Trans-Blot Turbo; 1705150; Bio-Rad, Hercules, CA, USA). The blocking and developing of the membranes were carried out as previously described [23]. Membranes were stripped after imaging vimentin and pan-keratin and exposed for 2 min to confirm that the membranes had been stripped, before incubating with GAPDH antibody as a loading control. Data shown are from three biological replicates in two technical replicates.

### 2.4. Immunoflourescence

The cells were immunostained as described previously and analysed under an inverted phase contrast microscope. All images were captured at the same automatic signal saturation threshold (AxioVert 40 CFL; Zeiss, Oberkochen, Germany) [19]. Images shown in the figures were auto-levelled in FIJI (version 1.54j) and are representative of three biological replicates in two technical replicates.

### 2.5. Live-Cell Imaging

The cells were seeded at 375 cells/well in slide chambers (94.6150.801; Sarstedt, Nümbrecht, Germany). After 24 h, DMEM was replaced with medium containing 5 ng/mL TGF-β1. Post-additional 72 h, the medium was replaced with DMEM with ALD-R491, or DMSO control and, 24 h thereafter, the medium was replaced with DMEM without phenol red, containing 0.05 μg/mL Hoechst (Sigma-Aldrich, Burlington, MA, USA) and ALD-R491 or DMSO. This was followed by live-cell imaging for 10 h with images taken every 20 min using the Mica Microhub Imaging System and LasX (Leica Microsystems, Wetzlar, Germany, software version 5.2.2). Images shown are auto-levelled in FIJI (version 1.54j). Data shown are from one biological replicate in two technical replicates.

### 2.6. Image Analysis

Cell shape was quantified by images taken at 0 and 10 h timepoints, using FIJI version v1.54j [24]. All cells with well-demarcated borders were manually outlined with the “Freehand selections” tool. The analysis of the correlation between migration speed and persistence was done in R (version 4.4.1), as shown in the Supplementary Text S1. Numbers of cells used for cell shape and spreading area analysis, control (N = 33), TGF-β1 (N = 11), and ALD-R491 (N = 17).

### 2.7. Tracking Cell Migration

Cell migration was tracked using the FIJI plug-in TrackMate tool [25]. The full documentation of this plug-in can be seen on the ImageJ wiki: https://imagej.net/plugins/trackmate/ (accessed 23 October 2024). The following specific settings were chosen for the tracking: detector, thresholding detector; intensity threshold, 10; simple LAP tracker: linking max distance, 100 µm; gap-closing max frame gap, 0; duration of tracks, 10 h (30 frames). All other settings remained at their default options. The tracks acquired were analysed using the in-built algorithms (https://imagej.net/plugins/trackmate/algorithms, accessed 23 October 2024. Numbers of cells used for migration, control (N = 48), TGF-β1 (N = 119), and ALD-R491 (N = 120) for cell migration.

### 2.8. Monitoring Nuclear Division

Nuclear division was monitored for 10 h by counting the cell nuclei using FIJI (version 1.54j). All well-demarcated and not-overlapping cell nuclei were manually outlined with the “Freehand selections” tool. Nuclei that underwent division and failed to divide, showing an “hourglass” nuclear shape, were counted. Numbers of total nuclei of cells used for cell division, control (N = 232), TGF-β1 (N= 101), and TGF-β1 + ALD R491 (N = 200). Two positions were used per condition. All images shown were auto-levelled in FIJI (version 1.54j).

### 2.9. Proteomic Analysis Using Label-Free Quantification Mass Spectrometry

Briefly, 5 mcg of protein from intermediate filament fraction were diluted to 1 M urea using 50 mM NH_4_HCO_3_ and processed as previously described [26,27]. Briefly, samples were reduced and alkylated by the sequential addition of 5 mM dithiothreitol (30 min, room temperature) and 15 mM iodoacetamide (20 min on ice) and, prior to a trypsin digestion ratio of 1:50 protein, desalted and analysed by a nano-LC–MS/MS. Peptide separation was achieved by reverse-phase HPLC with two mobile phase gradient system, using an C18 column (EASY-Spray PepMap RSLC; 50 cm × 75 μm ID, 2 μm; 40 °C; Thermo Fisher Scientific, Waltham, MA, USA) and a flow rate of 300 nl/min. Solvent A (0.1% formic acid in water) and solvent B (0.1% formic acid in 80% acetonitrile) at a 300 nL/min flow rate (RSLCnano HPLC system; Thermo Fisher Scientific, Waltham, MA, USA), with a gradient program of 0–5 min at 3% B, then increasing from 3% B to 50% B over the next 30 min. Mass spectrometry was performed using a Q Exactive HF hybrid quadrupole-Orbitrap (Thermo Fisher Scientific, Waltham, MA, USA). Data-dependent acquisition was performed with 10 product ion scans (centroid: resolution, 30,000; automatic gain control, 1 × 105 maximum injection time, 60 ms; isolation: normalized collision energy, 27; intensity threshold, 1 × 105) per full mass spectrometry scan (profile: resolution, 120,000; automatic gain control, 1 × 106; maximum injection time, 60 ms; scan range, 375–1500 *m/z*).

### 2.10. Protein Identification, Relative Quantification, Bioinformatic Functional Profiling and Interaction Analysis

Proteins were identified by searching the mass spectrometry data files against the Homo sapiens proteome database (www.uniprot.org/proteomes/UP000005640, downloaded 2 August 2021; 78,120 entries) using MaxQuant v. 1.6.4.0 with the label-free quantification (LFQ) and intensity-based absolute quantification (iBAQ) options selected [28,29,30]. Default settings were used with search parameters set to include the following modifications: carbamidomethyl-Cys (fixed); Met oxidation; protein N-terminal acetylation (variable); a maximum of two missed tryptic cleavages. Peptide-spectrum and protein identifications were filtered using a target-decoy approach at a false discovery rate (FDR) of 1%. Statistical analyses were performed using LFQ-Analyst (https://analyst-suite.monash-proteomics.cloud.edu.au/apps/lfq-analyst//, accessed 18 October 2021), where the LFQ intensity values were used for protein quantification [31]. Missing values were replaced by values drawn from a normal distribution of 1.8 standard deviations and a width of 0.3 for each sample (Perseus-type). Protein-wise linear models combined with empirical Bayesian statistics were used for differential expression analysis using the Bioconductor package *limma*, whereby the adjusted *p*-value cut-off was set at 0.05- and 2-fold change cut-off was set at 1. The Benjamini–Hochberg method of FDR correction was applied. The analysis of the relative amount of specific intermediate filament proteins in the enriched intermediate filament fractions was calculated using iBAQ values. This is a method for the calculation of the relative abundance of proteins in a dataset, which is achieved by dividing the total precursor intensities by the number of theoretically observable peptides in the protein. Proteins were relatively quantified by two or more unique peptides. We analysed three biological repeats in three technical triplicates, totalling nine samples. The g: Profiler tool (version e104_eg51_p15_3922dba) was used for functional enrichment analysis with the Benjamini–Hochberg FDR method applying a significance threshold of 0.05 [23]. The min and max size settings of the functional category were set to three and 500, respectively, with no electronic gene ontology (GO) annotations selected. GO terms for molecular function, biological process, cellular compartment, Kyoto Encyclopedia of Genes and Genomes (KEGG), and Reactome were assigned. The species was set to Homo sapiens. The data presented in this study are analysed using the same methods as previously described [32]. Protein interactors were determined with STRING 12 data resource. Signalling pathways were identified using the Signore 3.0., the SIGnalling Network Open Resource. If this resource lacked information on a protein, we used STRING data generated with the settings evidence and experiments, as indicated in the table.

### 2.11. Statistical Analysis

Statistical analysis was performed using the *ggpubr* and *rstatix* packages in R (version 4.4.1). The shape and migration parameters were compared using pairwise, two-sided *t*-tests. Figures/graphs were created using *ggplot2* and *patchwork* packages in R with a custom-made R script (Supplementary Text S1). Significance was determined at *p* ≤ 0.05. Pearson’s correlation between mean speed and persistence was calculated using linear regression. The Chi-Square Test with Yates’ Correction was used to determine differences in the proportions of abnormal nuclear features.

## 3. Results

### 3.1. ALD-R491 Partially Reverses the Phenotypes of the Epithelial-to-Mesenchymal Transition

**Figure 1:**
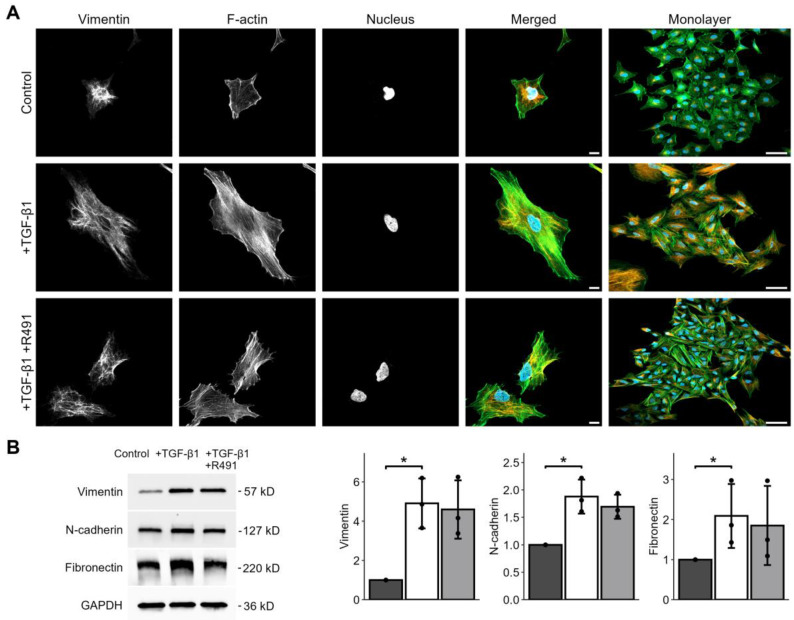
TGF-β1-induced cell and cytoskeletal EMT phenotypes in A549 lung cancer cells, with and without ALD-R491 treatment. A549 cells treated without or with TGF-β1, and subsequent ALD-R491 treatment (R491), with regards to (**A**) vimentin, F-actin, or nuclei, as indicated, with merged images showing vimentin (red), F-actin (green), and nuclei (blue). Scale bars 10 (**left**) and 50 µm (**right**). (**B**) Protein levels of EMT markers (**left**), as quantified (**right**) for control (dark grey), treatment with TGF-β1 (white), followed by ALD-R491 (light grey). Bar plots show mean, error bars standard deviation (SD), black dots the values of each biological repeat, *p* ≤ 0.05 (*). Representative cells are shown.

The intermediate filament protein vimentin regulates cell adhesion to the extracellular matrix, cell shape, and motility, all key for EMT-mediated cancer invasion [18]. To determine whether the vimentin-targeting drug ALD-R491 reverses EMT, we induced EMT in epithelial lung cancer cells with TGF-β1, followed by treatment with ALD-R491. TGF-β1-treated cells showed increased levels of EMT markers, such as vimentin, N-cadherin, and fibronectin, and increased formation of actin-stress fibres, all previously described markers of EMT. The ALD-R491 partially reversed these phenotypes of EMT (Figure 1A). The epithelial lung cancer cells showed minor, but detectable, levels of vimentin, with vimentin filaments mainly localised around the nucleus. TGF-β1 treatment resulted in a spatial re-organisation of vimentin towards the periphery of the cell (Figure 1A). Subsequent ALD-R491 treatment caused loss of wide actin stress fibres and a more central position of vimentin filaments around the nuclei. In line with our previous observations that ALD-491 increases the soluble fraction of vimentin more than three-fold, with no effect on the soluble fraction of actin, we observed a trend towards an increased filamentous actin–vimentin ratio in the ALD-R491-treated cells [19]. In line with our previous observation that ALD-R491 does not change total levels of vimentin in A549 cells, it did not change the total protein levels of the EMT markers in the cells (Figure 1B and Supplementary Figure S1) [21]. These observations suggest that ALD-R491 can reverse a subset of mesenchymal cytoskeletal and morphological phenotypes to epithelial.

**Figure 2:**
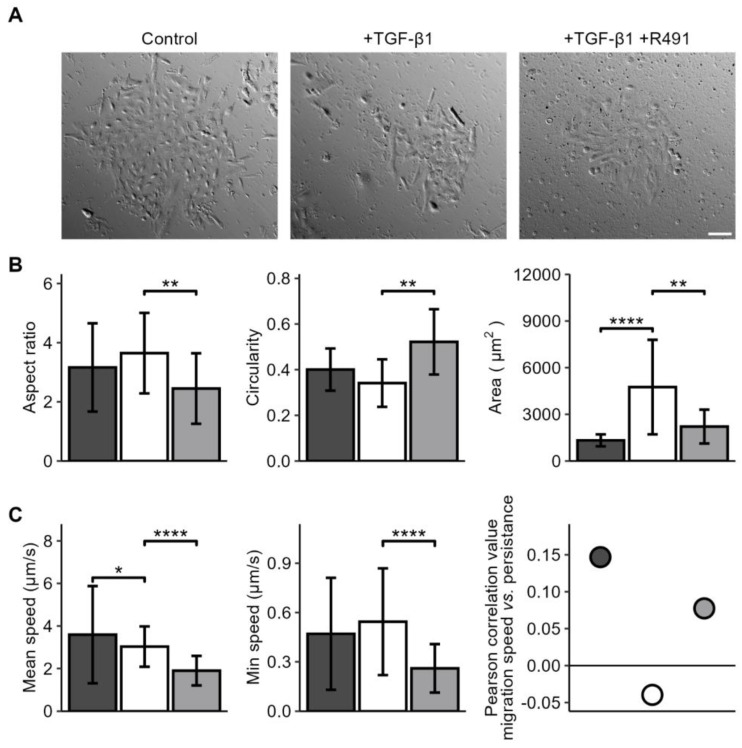
**ALD-R491 reverses EMT-dependent cell spreading and migration phenotypes to normal.** A549 lung cancer cell treated without (dark grey) or with TGF-β1 (white) and ALD-R491 (R491) (light grey), as indicated, shown in (**A**) cell monolayers, scale bar 100 µm, (**B**) cell aspect ratio, circularity, spreading area, and (**C**) cell migration speed, persistence and correlation between speed and persistence for control (dark grey), treatment with TGF-β1 (white), followed by ALD-R491 (light grey). Bar plots show mean, error bars standard deviation (SD), *p* ≤ 0.05 (*), *p* ≤ 0.01 (**), and *p* ≤ 0.0001 (****).

We then aimed to determine if vimentin dynamics is required for the changes of cell shape and motility that occur during EMT. For this, we induced EMT in the A549 lung cancer cells with TGF-β1, followed by ALD-R491 treatment, as described earlier. While the untreated A549 lung cancer cells displayed polygonal epithelial morphology, TGF-β1 -treated cells become less cohesive and slightly more elongated, a phenotype that was partly reversed by treatment with ALD-R491. TGF-beta treated cells became less round, a phenotype reversed by ALD-R491 treatment (Figure 2). The TGF-β1 treatment significantly increased the spreading area of the cells. TGF-β1 further increased the minimum cell speed of migration and changed the minor positive correlation between cell migration speed and migration directionality from positive to negative. These phenotypes were both reversed upon ALD-R491 treatment (Figure 2 and Supplementary Figure S2. Taken together, these findings suggest that the dynamics of vimentin filaments can contribute to the changes in cell shape and migration that accompany EMT.

**Figure 3:**
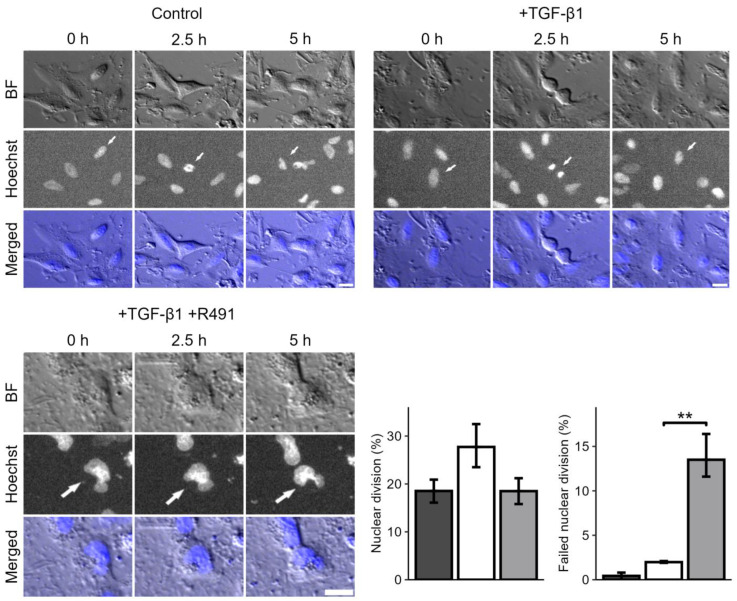
**Treatment with ALD-R491 and nuclear division during mitosis.** A549 lung cancer cells treated without (**top left**) and with TGF-β1 (**top right**), followed by ALD-R491 (R491) (**lower left**). Bright field images (BF), DNA (Hoechst) and merged images with BF and DNA (blue), are shown, as indicated. White arrows indicating dividing cells. Scalebar, 20 µm, with quantifications shown (**lower right**), with A549 treated without (dark grey) and TGF- β1 (white), followed by ALD-R491 (light grey), with regard to the proportion of nuclear division of all nuclei (left) and deformed, not-dividing proportion of dividing nuclei (right). Bar plots show mean, error bars, standard error of the mean (SEM), *p* ≤ 0.01 (**).

During the live-cell imaging of the cells, we further observed that ALD-R491-treated cells displayed a trend towards an abnormal division of their nuclei, as compared to the control. Many single cells failed to divide their nuclei, showing an hour-glass-shaped nuclei that remained deformed, or two nuclei. The DNA appeared more condensed in these deformed nuclei, as compared to the control (Figure 3). This suggests that the dynamic turnover of vimentin filaments can regulate the nuclear shape and division during mitosis.

### 3.2. EMT-Increased Binding of Extracellular Matrix, Cell Motility, Cytokinesis, Cytoskeletal, and RNA-Binding Proteins to Vimentin, Is Partially Reversed by ALD-R491

**Figure 4:**
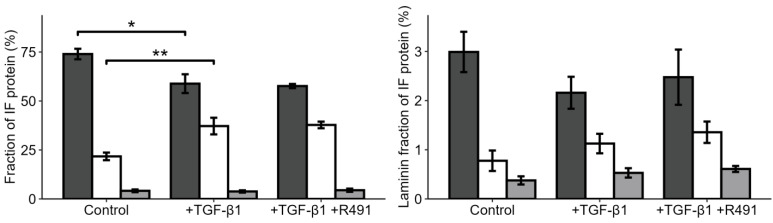
**TGF-β1 increases vimentin and reduces keratin in the intermediate filament fraction of the cytoskeleton**. The proportion of (**left**) keratin (dark grey), vimentin (white), and lamins (light grey) in the intermediate filament fraction, or of the (**right**) lamin A (dark grey), lamin B1 (white), and B2 (light grey) of all lamins in A549 lung cancer cell treated without (control) or with TGF-β1 and subsequent ALD-R491, as indicated. Bar plots show mean, error bars standard deviation (SD), *p* ≤ 0.05 (*), *p* ≤ 0.01 (**).

Vimentin has been proposed to act as a molecular scaffold that regulates intracellular signalling [33]. To gain insights into how EMT changes the relative proportion of intermediate filaments in epithelial lung cancer cells, and the proteins that bind to these filaments, we purified the intermediate filament fraction of the cytoskeleton in the cells, followed by mass spectrometry. Most intermediate filaments in the epithelial A594 cells were keratins, mainly keratin 1, 7, 8, and 18. However, low levels of vimentin were also detected (Figure 4, Supplementary Table S1). TGF-β1 increased the levels of vimentin in the intermediate filament fraction around two-fold, levels that did not change upon subsequent ALD-R491 treatment. The total levels of lamins and the ratio between variants of lamins were not significantly influenced by the ALD-R491 treatment (Figure 4, Supplementary Table S1).

**Figure 5:**
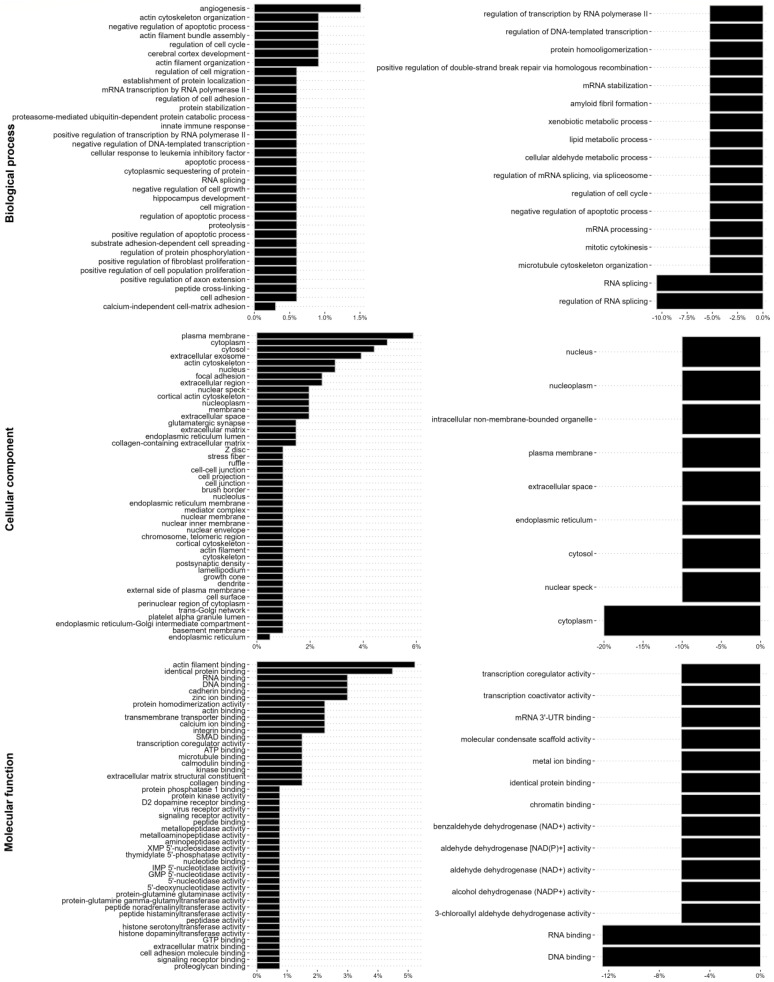
The EMT increases binding to the intermediate filaments of proteins that regulate the cell–extracellular matrix adhesion, cytoskeleton, cell shape, and cell motility, while ALD-R491 reduces binding of RNA metabolism and function. The changes of the vimentin interactome upon EMT (**left**) and by additional ALD-R491 treatment (**right**), shown as percentages, with biological function (**top**), cellular compartment (**middle**), and molecular function (**lower**), as indicated.

To determine if and how ALD-R491 can regulate the capacity of vimentin to control EMT, we characterised the protein interactome of the intermediate filament fraction of cells. For this, we identified and relatively quantified 838 proteins in the intermediate filament fraction, of which many were not previously known to bind to intermediate filaments (Supplementary Data Set S1). We observed that TGF-β1 changed the binding of 24 proteins to the intermediate filaments, of which all increased their levels (Table 1). Most of these EMT-induced intermediate filament-binding proteins were components or regulators of the ECM, cell–matrix adhesion, cytoskeleton, and cell migration (Table 1, Figure 5, Supplementary Table S2, Figures S3 and S4). The most pronounced change, with a fold change of 137, was for variants of fibronectin, which increased the binding upon EMT. These included Fibronectin 1 (FN1), Anastellin, Uhl-Y1, Ugl-Y2, and Ugl-Y3. Fibronectin promotes cell adhesion, spreading, and migration in health and disease [34,35]. The mass spectrometry method was based on the analysis of nine different samples, as detailed in the Materials and Methods section, with examples of the fibronectin results shown in Supplementary Figure S5. Proteins previously known to bind to fibronectin also showed an increased binding, i.e., Supervillin, which induces cell protrusions and regulates acto-myosin-based contractile force, and Transglutaminase-2, which stimulates integrin–fibronectin binding and TGF-β1 signalling [36,37,38,39]. We also observed increased levels in the additional proteins of TGF-β1 signalling, such as PML and TGF-β1 itself, and proteins known to induce cell migration, such as SON, Kinesin Family Member 4A, Aminopeptidase, Drebin-1, SUN2, Transmembrane protein 43, Caldesmon, and Spectrin [40,41,42,43,44,45,46,47,48,49]. Proteins known to regulate the contractile forces that cells use for cell migration were also identified, such as Drebin-1, SUN2, and Caldesmon, along with aminopeptidase, which regulates cell-ECM adhesion [43,45,50,51]. F-actin crosslinkers Alpha-actinin-1, Drebin-1, Spectrin, and Filamin A also increased; of these, Alpha-actinin-1 is required for EMT [44,49,52,53].

Additionally, we observed that the levels of proteins that process RNA, e.g., SON, and THR3, were increased in the intermediate fraction of cells upon TGF-β1 treatment (Table 1, and Supplementary Table S2). Taken together, these observations suggest that TGF-β1 significantly increases the binding to intermediate filaments by proteins that contributes to EMT through ECM-cell adhesion, cytoskeletal organisation, and cell migration.

To determine if ALD-R491 can revert the EMT-associated intermediate filament interactome to normal, we compared it between TGF-β1-treated cells that subsequently were treated with ALD-R491 or DMSO control. We observed that the ALD-R491 reversed the TGF-β1-induced binding of RNA splicing proteins to the intermediate filaments (Table 2, Figure 5, Supplementary Figures S3 and S4). These included the binding of the protein SON, which was reduced to levels prior to TGF-β1 treatment (Table 2). SON is an mRNA-splicing cofactor that is required for mitosis and cell migration [41,54]. We also observed reduced binding to the intermediate filaments of the proteins FUS and Aldehyde dehydrogenase, ALDH3A1. FUS is part of the hnRNP complex and supports the stability of pre-mRNA, mRNA export, and stability [55,56,57,58]. The TGF-β1 treatment increased the proportion of these two proteins, although at a fold-change that was slightly lower than the two-fold cut-off value we used. Taken together, these findings suggest that the ALD-R491-mediated inhibition of vimentin dynamics suppresses the spatial and timely localisation of proteins to the subcellular site required for dynamic cytoskeletal, mechanical, morphological, and motile changes.

We further analysed changes in the intermediate filament interactome regarding biological process, cellular compartment, and molecular mechanisms. TGF-β1 induced the binding of proteins previously known to localise to the plasma membrane and cytoskeleton of cells, as well as to cell–extracellular matrix adhesions, extracellular space, and the ECM, including the collagen-containing extracellular matrix, and proteins with a molecular function in the cytoskeleton, extracellular matrix, or cell adhesion (Figure 5). In addition, we observed a lower, but still significantly increased, binding to proteins with previously described function in RNA splicing and RNA metabolism, as well as nuclear proteins with DNA binding properties, including the mRNA-splicing protein SON (Figure 5 and Supplementary Table S2). Further treatment with ALD-R491 resulted in a decreased binding of proteins linked to RNA binding and RNA splicing, including SON, protein scaffolding and aldehyde dehydrogenase (Figure 5, Supplementary Figure S4 and Table S3). Four of the interactome proteins were previously reported to interact with each other (Supplementary Figure S6). Taken together, this highlights that vimentin filaments can regulate EMT via different signalling pathways and cellular functions.

## 4. Discussion

In this study, we show that ALD-R491, a small molecular compound that specifically increases the stability and suppresses the dynamic exchange of units within vimentin intermediate filaments, partially reverses the EMT phenotypes of human lung cancer cells. Specifically, the TGF-β1-induced mesenchymal phenotypes of cell spreading and migration, as well as mesenchymal distribution or the cytoskeleton, were reversed to epithelial with no change in the protein levels of known EMT markers. These apparently conflicting observations can be reconciled if we acknowledge that EMT programs are not binary switches in which cells are either in epithelial or mesenchymal states. It remains unclear if there are discrete, definable stages along the EMT spectra, or, rather, if it is a continuum. Cancer-associated EMT is often only partially or transiently activated, and end-stage, mesenchymal markers are therefore considered to be uninformative for cancer [2]. Therefore, for cancer, the partial reversion of a subset of EMT phenotypes that we observe can be just as, or even more relevant, for clinical practice, as a full EMT. In an extensive, global proteomic analysis of all proteins in the intermediate filament fraction of cells, we identified 838 proteins, of which many were not previously known to bind to intermediate filaments. We observed that EMT increased the fraction of vimentin within the intermediate filaments of cells, and the binding to components and regulators of the ECM, cytoskeleton, and cell motility. It also, to a lesser extent, increased binding to RNA-binding proteins. An important focus for future studies would be to determine if the changes in the intermediate filament interactome can be linked to any different histological features of lung cancer tissue. ALD-R491 primarily reduced the binding of RNA-binding proteins. The finding that ALD-R491 treatment reduced the spreading area of the cells is in line with previous observations that the spatial distribution of vimentin filaments governs cell spreading [6,59]. The observation that ALD-R491 reduced cell migration speed and persistence in the TGF-β1-treated lung cancer cells is consistent with previous findings that the dynamic turnover of vimentin filaments promotes cell migration ex vivo and in vivo, and that vimentin is required for the invasion of lung cancer into the surrounding tissues [4,10,60]. Our findings are in line with earlier observation that, in addition to the protein levels, the dynamic properties of vimentin filaments are also important for cell motility. For example, vimentin re-organization towards the cell periphery by p21-activated kinase (PAK) increases cell migration, whereas oncogenes increase the soluble pool of vimentin, redistributes vimentin towards the nucleus, and induces cell invasion [61,62]. We have further observed that ALD-R491 reduced the cell migration speed of fibroblast cells in a vimentin-dependent manner [19]. The finding that ALD-R491 reversed the EMT-induced negative correlation between the persistence and speed of cell migration to positive provides novel insights into the differences between epithelial and mesenchymal cell migration and highlights that vimentin dynamics can be a main factor in the switch between EMT and these two separate types of cell migration. We observed that EMT mainly increased binding to the intermediate filament fraction of components and regulators of the ECM, cell adhesion, cell cytoskeleton, and cell motility. This can be an indirect consequence that the transcription and synthesis of these proteins are significantly increased when cells acquire motile properties during EMT. However, for fibronectin, where we observed an almost 140-fold increase in binding to intermediate filaments, total fibronectin levels only increased two-fold; therefore, it is also possible that binding to vimentin acts to regulate the local distribution and deposition of fibronectin into the ECM. This would be like a recently proposed binding and/or buffering function of vimentin for the regulation of the deposition of integrin in the organisation of cell–matrix adhesions [63,64]. Fibronectin is known to significantly increase cell adhesion signalling, migration, and invasion, and to contribute to EMT in cancer [65]. TGF-β1 further induced the intermediate filament binding of the protein Transglutaminase-2 (TGM2), an ECM component which stabilises the ECM by strengthening the interaction between integrins and fibronectin and stimulates TGF-β1-signalling [36,37,38]. Taken together with the essential role of vimentin in producing collagen I, via stabilisation of collagen mRNA, these observations suggest that vimentin can regulate EMT by different ECM-regulating pathways [17]. The possible effect of ALD-R491 on the division of the nucleus during cytokinesis is consistent with earlier finding that vimentin maintains nuclear shape and mechanical stability, and that vimentin dynamics contributes to successful mitosis [66,67]. While EMT induced the binding of the protein SON to vimentin, ALD-R491 reversed this binding back to normal levels in epithelial cells. SON is an RNA-binding protein which promotes the splicing of many transcripts which possess weak splice sites, such as AURKB, PCNT, and AKT1, which all function in cytokinesis [41]. SON is required for microtubule dynamics, spindle pool separation during mitosis, and the proliferation of epithelial cells, and it is further linked to poor cancer prognosis [41,47,68]. We also observed a reversion of TGF-β1-increased binding to vimentin in FUS and ALDH3A1, although the increases were under the two-fold cut-off that we used for the data. FUS is an RNA-binding protein, which is overexpressed in non-small cell lung cancer (NSCLC) tissue, reducing E-cadherin levels in NSCLC cells, and it is linked to poor patient prognosis [69]. ALDH3A1 is an aldehyde dehydrogenase required for cell migration and the invasion of A549 lung cancer cells [70]. Taken together, this suggests SON, FUS, and ALDHA3 can be potential targets for the treatment of cancer-associated EMT. Vimentin filaments have previously been found to bind to proteins that stabilise mRNA, regulate mRNA splicing, and stimulate protein production, such as ribosomal proteins, and has also been found to assemble in clusters where ribosomes are enriched [27,71]. The presence of RNA-binding proteins highlights the possibility that vimentin-facilitated RNA metabolism and processing is instrumental for EMT, like the role RNA processing plays in EMT in embryonic development [71].

Taken together, our data are in line with earlier reports that vimentin filaments act as a change-platform that allows spatially localised protein production when cells transiently change shape, such as during EMT-induced cell migration and in cytokinesis. It further suggests that the intact dynamic turnover of vimentin filaments is essential for major changes in cell shape during cell migration and cytokinesis, as well as for EMT. EMT and cell motility are fundamental processes in the progression and invasion of carcinomas, such as epithelial-derived lung cancer, because EMT provides the cells with the mechanical, adhesive, and motile properties that cause them to invade and metastasise into the surrounding tissue [6]. Our observation that the drug ALD-R491 partially reverses the EMT phenotypes of lung cancer cells suggests that the drug could be used to meet the urgent need to identify novel tools to suppress EMT. The finding that the ALD-R491 drug partially reverses the cell phenotypes of the EMT in lung cancer cells points to the possibility of using ALD-R491 to develop future treatment against lung cancer. It could offer opportunities to overcome EMT-linked resistance mechanisms in Osimertinib-treated lung cancer patients, an urgent clinical issue [11]. The identification of novel intermediate filament-binding proteins, and how these change during EMT and upon the addition of ALD-R491, increases our understanding of how vimentin dynamics can regulate EMT, cell invasion, and cancer metastasis.

Taken together, our findings highlight a large number of potential novel interactors with intermediate filaments and are in line with the hypothesis that the binding to vimentin of ECM and cell migration proteins, as well as of the proteins of RNA metabolism and function, such as the protein SON, allows the function of molecular pathways at the time and space required for dynamic changes of cells, such as during EMT and cancer metastasis. These observations expand our understanding of the functions of vimentin in cancer metastasis.

## Figures and Tables

**Table 1:** Changes in protein levels for proteins altered in the vimentin interactome of TGFβ1-treated A549 cells relative to the native epithelial A549 cells, protein and gene names, protein ID, signalling pathway, fold change, and *p*-values are shown, as indicated.

Protein	Gene	Protein ID	Signalling Pathway	TGFβ1 vs. A549 Foldchange (*p*-Value)
Fibronectin;Anastellin;Ugl-Y1;Ugl-Y2;Ugl-Y3	*FN1*	P02751	Integrin, Collagen, Fibrin	137.19 (3.09 × 10^−13^)
Transforming growth factor-β1-induced protein ig-h3	*TGF-Β1*	Q15582	TGF-Β1, MAPK	28.05 (1.33 × 10^−10^)
Protein-glutamine gamma-glutamyltransferase 2	*TGM2*	P21980	FN1, SPFN1, HSPB6	17.63 (2.09 × 10^−18^)
5-nucleotidase	*NT5E*	P21589	AMP- NAD- nucleotides	6.92 (4.91 × 10^−12^)
Aminopeptidase N	*ANPEP*	P15144	an aminopeptidase	5.58 (1.06 × 10^−7^)
Neurabin-2	*PPP1R9B*	Q96SB3	F-actin, Rac, Dopamine D1	4.92 (0.000159)
Zinc finger protein 185	*ZNF185*	O15231	* Wnt	4.86 (4.95 × 10^−10^)
Drebrin 1	*DBN1*	Q16643	* F-actin	4.32 (1.88 × 10^−10^)
Caldesmon	*CALD1*	E9PGZ1	F-actin,, myosin, calmodulin	4.08 (5.49 × 10^−10^)
SUN-domain-containing protein 2	*SUN2*	Q9UH99	LINC complex	3.92 (0.000905)
SON	*SON*	P18583	TUBG1, KATNB1, AURKB	3.78 (0.0034)
CTP synthase 1	*CTPS1*	A0A3B3IRI2	CTP	3.68 (3.42 × 10^−5^)
Bcl-2-associated transcription factor 1	*BCLAF1*	A0A3B3ITZ9	CCND1 mRNA	3.48 (0.00372)
Supervillin	*SVIL*	A0A6I8PIX7	F-actin	3.23 (0.000905)
Protein PML	*PML*	P29590	PML-NBs	3.18 (0.000565)
Protein disulfide-isomerase A4	*PDIA4*	A0A499FI48	* HSP, ERO1	2.85 (0.000398)
Transmembrane protein 43	*TMEM43*	Q9BTV4	RNF26	2.62 (1.04 × 10^−6^)
Uncharacterised protein C17orf85	*C17orf85*	Q53F19	mRNA	2.58 (0.00418)
Thyroid-hormone-receptor-associated protein 3	*THRAP3*	A0A3B3ITZ9	mRNA, DNA	2.36 (0.00372)
Alpha-actinin-1	*ACTN1*	P12814	F-actin	2.25 (3.66 × 10^−10^)
LIM domain and actin-binding protein 1	*LIMA1*	Q9UHB6	F-actin	2.22 (0.000905)
Kinesin-like protein 14	*KIF14*	Q15058	Tubulin, CDKN1B	2.11 (0.00102)
Spectrin alpha chain, non-erythrocytic 1	*SPTAN1*	Q13813	F-actin, Calmodulin	2.06 (0.00151)
Filamin-A	*FLNA*	P21333	F-actin, SEMA3A	2.01 (9.05 × 10^−6^)

* from STRING.

**Table 2:** Significantly changed proteins in vimentin interactome of TGFβ1- and ALD-R491-treated A549 cells, relative to TGFβ1-treated A549 cells, protein and gene names, protein ID, signalling pathway, fold-change, and *p*-values are shown, as indicated.

Protein	Gene	Protein ID	Signaling Pathway	TGFβ1 vs. A549 Fold Change (*p*-Value)
Protein SON	*SON*	P18583	TUBG1, KATNB1, AURKB	−4.89 (0.00156)
Aldehyde dehydrogenase, dimeric NADP-preferring	*ALDH3A1*	P30838	Aldehyde substrates	−2.62 (0.00257)
RNA-binding protein FUS	*FUS*	P35637	mRNA, DNA	−2.62 (0.00354)

## Supplementary Materials

The following supporting information can be downloaded at: https://www.mdpi.com/article/10.3390/cancers17010081/s1, Figure S1: Representative Western blot images (top) showing original blots, all bands, and molecular weight markers, in kDa, as indicated (lower panel). Figure S2: Cell migration of lung cancer cell line A549. Maximum, minimum, median and mean speed, as indicated. Control (dark grey), TGF-β1 -treatment (light grey) and drug treatment (grey). Bar graphs show mean, error bars standard deviation (SD), *p* ≤ 0.05 (*), *p* ≤ 0.01 (**), *p* ≤ 0.001 (***), and *p* ≤ 0.0001 (****). Table S1: The fraction of each intermediate filament protein in the intermediate filament fraction of A549 cells, treated without (Control), and with TGF-β1 without (+TGF-β1), or with sequential treatment with ALD-R491 (+R491). The fraction of each protein of total (%), and each sample from three biological repeats, as indicated. Table S2: The Gene ontology for EMT-induced binding to intermediate filaments, as indicated. Figure S3: EMT increases the binding to intermediate filaments of proteins that regulate the cell-extracellular matrix adhesion, cytoskeleton, cell shape and cell motility, while ALDR491 reduces binding of RNA metabolism and function. The full list of GO annotations of the vimentin interactome upon treatment with TGF-β1 (A) or additional ALD-R491 (B), shown as percentages, with the Biological function (left), Cellular compartment (top right), and Molecular activity (bottom right panels), as indicated. Figure S4: EMT increases, while ALD-R491 decreases, the binding of proteins that regulate RNA metabolism and function to intermediate filaments. GO annotations of changes of vimentin interactome of lung cancer cells upon treatment with TGF-β1 (left), or between TGF-β1 only and TGF-β1 with additional ALD-R491 (right panel), shown as percentages, with the Biological function (top), Cellular compartment (middle), and Molecular activity (lower panels), as indicated. Figure S5: Example of Mass Spectrometry-based quantitative proteomic data. Each of the three, separate, biological replicates were divided and analysed in three technical replicates. The nine samples were analysed, and peptides identified, as outlined in the material and methods section. The examples show the levels of fibronectin 1 peptides identified in the analysis, in the nine samples, as indicated. Black dot indicates imputed value (absent in this example). Table S3: The Gene ontology for EMT-induced vimentin binding proteins which were changed upon treatment with ALD-R491, with regards to Biological process, Cellular component and Molecular function, as indicated. Figure S6: STRING analysis of protein-to-protein interactions between proteins of the vimentin interactome. The changes of the vimentin interactome upon TGF-β1-induced EMT (left), and upon additional treatment with ALD-R491 (right). Supplementary Document S1 and Supplementary Data Set S1.
