# Supplementary material for: The Vimentin-Targeting Drug ALD-R491 Partially Reverts the Epithelial-to-Mesenchymal Transition and Vimentin Interactome of Lung Cancer Cells
Source: Cancers (Basel). 2024 Dec 30;17(1):81. doi: 10.3390/cancers17010081 (PMC11720119; doi:10.3390/cancers17010081)
Supplement: Supplementary file 1 [file cancers-17-00081-s001.zip › Supplementary Document 1.pdf]

Supplementary material

Supplementary Text 1. Script in R for the correlation between the speed and persistence of cell migration.

```
correlation.data <-
remove.outliers(remove.outliers(graphical.data,'PERSISTANCE'),'TRACK_MEAN_SPEED')

conditions <- unique(graphical.data[,WELL])

for(i in 1:length(conditions))

{

  c <- cor.test(correlation.data[WELL==conditions[i]]$PERSISTANCE,
correlation.data[WELL==conditions[i]]$TRACK_MEAN_SPEED, method='pearson')

  coeff <- c(coeff, unlist(c$estimate))

}

coeff.data <- data.table(coefficients=unlist(coeff), WELL=conditions)

 correlation <- ggplot(coeff.data,aes(WELL, coefficients, label=round(coefficients,2))) +
geom_point(aes(WELL, coefficients, color = WELL), size = 12)+ geom_text(aes(WELL, coefficients),
color = c('white','black','black')) + ylab('Correlation coefficents') + xlab(' ') + theme_classic() +
scale_colour_manual(values = c('#3b3c3c','#b0b0b3','#939293')) + guides(color='none') + theme(axis.title
= element_text(face = 'bold'), axis.text.y = element_text(face = 'bold', colour = 'black'), axis.text.x =
element_text(face = 'bold', colour = 'black')) + scale_y_continuous(limits = c(-0.1,0.2))
```

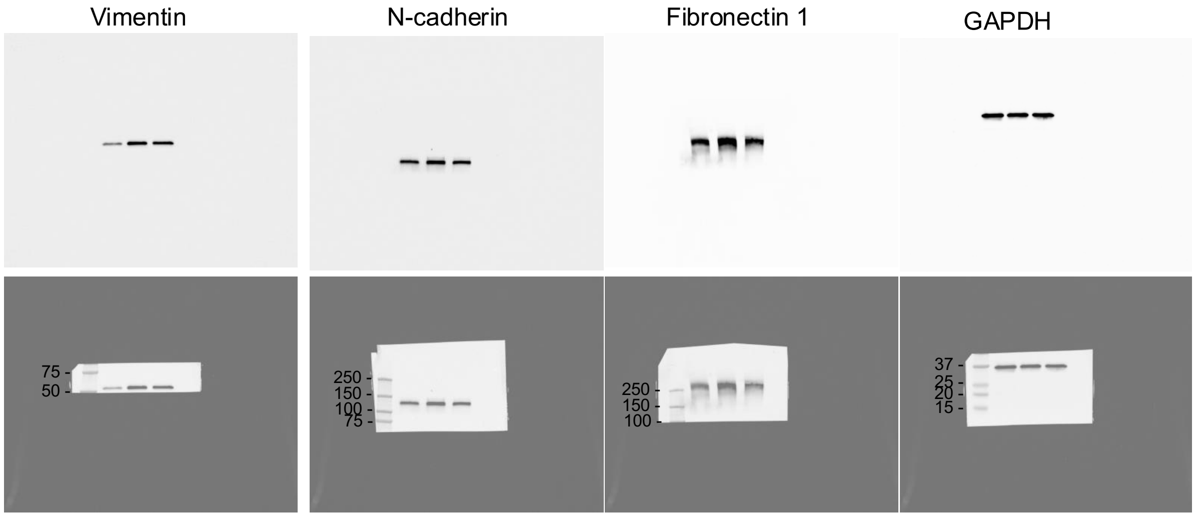

Supplementary Figure 1. Representative Western blot images (top) showing original blots, all bands, and molecular weight markers, in kDa, as indicated (lower panel).

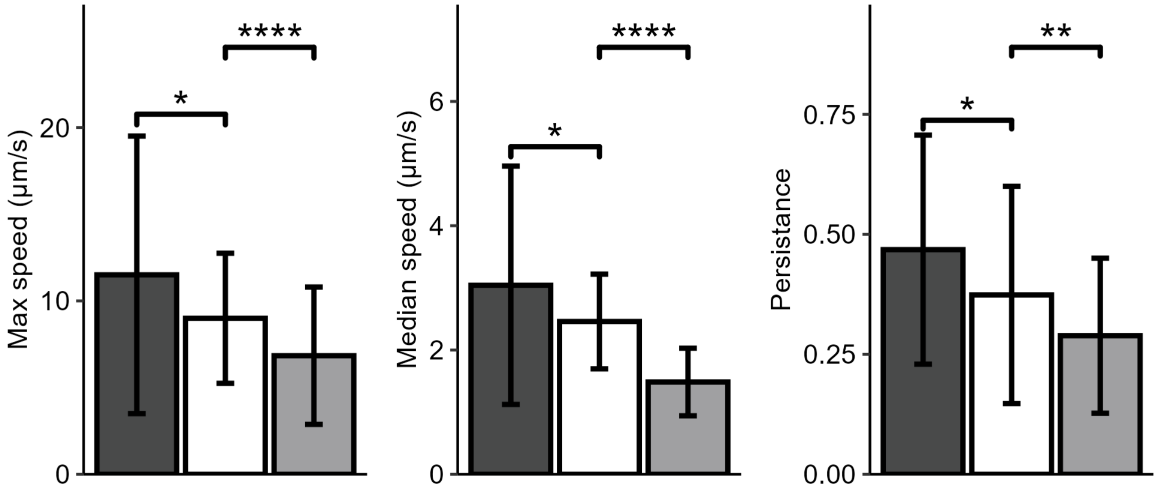

Supplementary Figure 2. Cell migration of lung cancer cell line A549. Maximum, minimum, median and mean speed, as indicated. Control (dark grey), TGF-β1 -treatment (light grey) and drug treatment (grey). Bar graphs show mean, error bars standard deviation (SD), $p \leq 0.05$ (*), $p \leq 0.01$ (**), $p \leq 0.001$ (***), and $p \leq 0.0001$ (****).

Supplementary Table 1. The fraction of each intermediate filament protein in the intermediate filament fraction of A549 cells, treated without (Control), and with TGF-β1 without (+TGF-β1), or with sequential treatment with ALD-R491 (+R491). The fraction of each protein of total (%), and each sample from three biological repeats, as indicated.

	Fraction of total intermediate filament proteins (%)								
	Control			TGF-β1			R491		
Protein (Gene name)	1	2	3	1	2	3	1	2	3
Vimentin (VIM)	19.91	21.47	23.77	41.93	33.7	35.97	38.6	35.83	38.9
Keratin 1 (KRT1)	1.79	2	1.25	1.63	1.18	4.63	2.27	1.32	1.57
Keratin 5 (KRT5)	0.06	0.09	0.05	0.07	0.05	0.15	0.53	0.05	0.07
Keratin 6A (KRT6A)	0.03	0.06	0.02	0.06	0.02	0.04	0.02	0.02	0.05
Keratin 7 (KRT7)	8.57	11.39	10.93	8.54	8.65	6.73	7.41	6.58	6.99
Keratin 8 (KRT8)	31.02	27.74	28.08	16.89	24.55	19.57	19.49	21.2	20.64
Keratin 9 (KRT9)	0.73	1.17	0.63	0.87	0.65	4.95	1.24	0.66	1.16
Keratin 10 (KRT10)	1.05	1.11	0.73	0.9	0.66	1.19	1.68	1.11	0.75
Keratin 14 (KRT14)	0.06	0.08	0.04	0.07	0.05	0.12	0.06	0.03	0.07
Keratin 18 (KRT18)	33.16	30.68	29.4	24.43	26.74	23.19	24.93	27.72	25.27
Lamin A (LMNA)	2.59	2.97	3.41	2.48	2.17	1.83	1.89	3.01	2.53
Lamin B1 (LMNB1)	0.61	0.71	1.01	1.35	0.97	1.06	1.18	1.6	1.29
Lamin B2 (LMNB2	0.31	0.35	0.47	0.64	0.48	0.47	0.57	0.68	0.58
Alpha internexin (INA)	0.06	0.11	0.1	0.08	0.06	0.05	0.1	0.09	0.07
Nestin (NES)	0	0	0.01	0.01	0	0	0	0	0
Synemin (SYNM)	0.02	0.05	0.08	0.05	0.05	0.02	0.01	0.08	0.04

Supplementary Table 2. The Gene ontology for EMT-induced binding to intermediate filaments, as indicated.

Protein	Biological process	Cellular component	Molecular function
FN1	acute-phase response [GO:0006953]; angiogenesis [GO:0001525]; biological process involved in interaction with symbiont [GO:0051702]; calcium-independent cell-matrix adhesion [GO:0007161]; cell adhesion [GO:0007155]; cell-matrix adhesion [GO:0007160]; cell-substrate junction assembly [GO:0007044]; endodermal cell differentiation [GO:0035987]; heart	apical plasma membrane [GO:0016324]; basement membrane [GO:0005604]; blood microparticle [GO:0072562]; collagen-containing extracellular matrix [GO:0062023]; endoplasmic reticulum lumen [GO:0005788]; endoplasmic reticulum-Golgi intermediate compartment [GO:0005793]; extracellular exosome [GO:0070062];	collagen binding [GO:0005518]; extracellular matrix structural constituent [GO:0005201]; heparin binding [GO:0008201]; identical protein binding [GO:0042802]; integrin binding [GO:0005178]; peptidase activator activity [GO:0016504]; protease binding [GO:0002020]; proteoglycan binding

	development [GO:0007507]; integrin activation [GO:0033622]; integrin-mediated signaling pathway [GO:0007229]; negative regulation of monocyte activation [GO:0150102]; negative regulation of transforming growth factor beta production [GO:0071635]; nervous system development [GO:0007399]; neural crest cell migration involved in autonomic nervous system development [GO:1901166]; peptide cross-linking [GO:0018149]; positive regulation of axon extension [GO:0045773]; positive regulation of cell population proliferation [GO:0008284]; positive regulation of fibroblast proliferation [GO:0048146]; positive regulation of gene expression [GO:0010628]; positive regulation of phosphatidylinositol 3-kinase/protein kinase B signal transduction [GO:0051897]; positive regulation of substrate-dependent cell migration, cell attachment to substrate [GO:1904237]; regulation of cell shape [GO:0008360]; regulation of ERK1 and ERK2 cascade [GO:0070372]; regulation of protein phosphorylation [GO:0001932]; response to wounding [GO:0009611]; substrate adhesion-dependent cell spreading [GO:0034446]; wound healing [GO:0042060]	extracellular matrix [GO:0031012]; extracellular region [GO:0005576]; extracellular space [GO:0005615]; fibrinogen complex [GO:0005577]; plasma membrane [GO:0005886]; platelet alpha granule lumen [GO:0031093]	[GO:0043394]; signaling receptor binding [GO:0005102]
TGFBI	angiogenesis [GO:0001525]; cell adhesion [GO:0007155]; cell population proliferation [GO:0008283]; chondrocyte differentiation [GO:0002062]; extracellular matrix organization [GO:0030198]; localization [GO:0051179]; negative regulation of cell adhesion [GO:0007162]; response to stimulus [GO:0050896]; visual perception [GO:0007601]	basement membrane [GO:0005604]; collagen-containing extracellular matrix [GO:0062023]; extracellular exosome [GO:0070062]; extracellular matrix [GO:0031012]; extracellular region [GO:0005576]; extracellular space [GO:0005615]; plasma membrane [GO:0005886]; trans-Golgi network [GO:0005802]	cell adhesion molecule binding [GO:0050839]; collagen binding [GO:0005518]; extracellular matrix binding [GO:0050840]; extracellular matrix structural constituent [GO:0005201]; identical protein binding [GO:0042802]; integrin binding [GO:0005178]
NT5E	adenosine biosynthetic process [GO:0046086]; ADP catabolic process [GO:0046032]; AMP catabolic process [GO:0006196]; ATP metabolic process [GO:0046034]; calcium ion homeostasis	cell surface [GO:0009986]; cytosol [GO:0005829]; external side of plasma membrane [GO:0009897]; extracellular exosome [GO:0070062]; membrane [GO:0016020]; nucleoplasm	5'-deoxynucleotidase activity [GO:0002953]; 5'-nucleotidase activity [GO:0008253]; GMP 5'-nucleotidase activity [GO:0050484]; identical protein binding [GO:0042802]; IMP 5'-

	[GO:0055074]; DNA metabolic process [GO:0006259]; inhibition of non-skeletal tissue mineralization [GO:0140928]; leukocyte cell-cell adhesion [GO:0007159]; negative regulation of inflammatory response [GO:0050728]; response to ATP [GO:0033198]; response to inorganic substance [GO:0010035]	[GO:0005654]; plasma membrane [GO:0005886]	nucleotidase activity [GO:0050483]; nucleotide binding [GO:0000166]; thymidylate 5'-phosphatase activity [GO:0050340]; XMP 5'-nucleosidase activity [GO:0106411]; zinc ion binding [GO:0008270]
ANPEP	angiogenesis [GO:0001525]; cell differentiation [GO:0030154]; peptide catabolic process [GO:0043171]; proteolysis [GO:0006508]	cytoplasm [GO:0005737]; endoplasmic reticulum-Golgi intermediate compartment [GO:0005793]; external side of plasma membrane [GO:0009897]; extracellular exosome [GO:0070062]; extracellular space [GO:0005615]; lysosomal membrane [GO:0005765]; plasma membrane [GO:0005886]; secretory granule membrane [GO:0030667]	aminopeptidase activity [GO:0004177]; metalloaminopeptidase activity [GO:0070006]; metallopeptidase activity [GO:0008237]; peptide binding [GO:0042277]; signaling receptor activity [GO:0038023]; virus receptor activity [GO:0001618]; zinc ion binding [GO:0008270]
PPP1R9B	actin filament depolymerization [GO:0030042]; actin filament organization [GO:0007015]; calcium-mediated signaling [GO:0019722]; cell migration [GO:0016477]; cellular response to epidermal growth factor stimulus [GO:0071364]; cellular response to estradiol stimulus [GO:0071392]; cellular response to morphine [GO:0071315]; cellular response to peptide [GO:1901653]; cellular response to xenobiotic stimulus [GO:0071466]; cerebral cortex development [GO:0021987]; dendrite development [GO:0016358]; developmental process involved in reproduction [GO:0003006]; filopodium assembly [GO:0046847]; hippocampus development [GO:0021766]; learning [GO:0007612]; male mating behavior [GO:0060179]; negative regulation of cell growth [GO:0030308]; neuron projection development [GO:0031175]; positive regulation of protein localization to actin cortical patch [GO:1904372]; positive regulation of protein localization to plasma membrane [GO:1903078]; protein localization to actin cytoskeleton [GO:1903119]; protein localization to cell periphery [GO:1990778]; regulation of cell	actin cytoskeleton [GO:0015629]; adherens junction [GO:0005912]; cortical actin cytoskeleton [GO:0030864]; cytoplasm [GO:0005737]; cytoplasmic side of dendritic spine plasma membrane [GO:1990780]; dendrite [GO:0030425]; dendritic spine head [GO:0044327]; dendritic spine neck [GO:0044326]; filopodium [GO:0030175]; growth cone [GO:0030426]; lamellipodium [GO:0030027]; neuronal cell body [GO:0043025]; nucleoplasm [GO:0005654]; plasma membrane [GO:0005886]; postsynaptic density [GO:0014069]; protein phosphatase type 1 complex [GO:0000164]; ruffle membrane [GO:0032587]	actin filament binding [GO:0051015]; D2 dopamine receptor binding [GO:0031749]; kinase binding [GO:0019900]; protein kinase activity [GO:0004672]; protein phosphatase 1 binding [GO:0008157]; protein phosphatase inhibitor activity [GO:0004864]; transmembrane transporter binding [GO:0044325]

	cycle [GO:0051726]; regulation of cell growth by extracellular stimulus [GO:0001560]; regulation of cell population proliferation [GO:0042127]; regulation of exit from mitosis [GO:0007096]; regulation of opioid receptor signaling pathway [GO:2000474]; regulation of protein phosphorylation [GO:0001932]; reproductive system development [GO:0061458]; response to amphetamine [GO:0001975]; response to immobilization stress [GO:0035902]; response to kainic acid [GO:1904373]; response to L-phenylalanine derivative [GO:1904386]; response to nicotine [GO:0035094]; response to prostaglandin E [GO:0034695]; response to steroid hormone [GO:0048545]; RNA splicing [GO:0008380]		
ZNF185	_	cytoplasm [GO:0005737]; cytoskeleton [GO:0005856]; focal adhesion [GO:0005925]	zinc ion binding [GO:0008270]
DBN1	actin filament organization [GO:0007015]; cell communication by chemical coupling [GO:0010643]; cell communication by electrical coupling [GO:0010644]; cytoplasmic sequestering of protein [GO:0051220]; in utero embryonic development [GO:0001701]; maintenance of protein location in cell [GO:0032507]; neural precursor cell proliferation [GO:0061351]; neuron projection morphogenesis [GO:0048812]; positive regulation of axon extension [GO:0045773]; positive regulation of dendritic spine morphogenesis [GO:0061003]; positive regulation of receptor localization to synapse [GO:1902685]; positive regulation of synaptic plasticity [GO:0031915]; postsynaptic actin cytoskeleton organization [GO:0098974]; regulation of actin filament polymerization [GO:0030833]; regulation of dendrite development [GO:0050773]; regulation of	actin cytoskeleton [GO:0015629]; actin filament [GO:0005884]; actomyosin [GO:0042641]; cortical actin cytoskeleton [GO:0030864]; cortical cytoskeleton [GO:0030863]; cytoplasm [GO:0005737]; cytoskeleton [GO:0005856]; dendrite [GO:0030425]; gap junction [GO:0005921]; glutamatergic synapse [GO:0098978]; growth cone [GO:0030426]; lamellipodium [GO:0030027]; postsynaptic cytosol [GO:0099524]; postsynaptic density [GO:0014069]; postsynaptic membrane [GO:0045211]	actin binding [GO:0003779]; actin filament binding [GO:0051015]; cadherin binding [GO:0045296]; profilin binding [GO:0005522]

	neuronal synaptic plasticity [GO:0048168]		
CALD1	actin filament bundle assembly [GO:0051017]; angiogenesis [GO:0001525]; muscle contraction [GO:0006936]	actin cytoskeleton [GO:0015629]	actin binding [GO:0003779]; calmodulin binding [GO:0005516]; myosin binding [GO:0017022]
SUN2	centrosome localization [GO:0051642]; meiotic cell cycle [GO:0051321]; mitotic spindle organization [GO:0007052]; nuclear matrix anchoring at nuclear membrane [GO:0090292]; nuclear migration [GO:0007097]; nuclear migration along microfilament [GO:0031022]; nucleokinesis involved in cell motility in cerebral cortex radial glia guided migration [GO:0021817]; positive regulation of cell migration [GO:0030335]	chromosome, telomeric region [GO:0000781]; condensed nuclear chromosome [GO:0000794]; endosome membrane [GO:0010008]; meiotic nuclear membrane microtubule tethering complex [GO:0034993]; nuclear envelope [GO:0005635]; nuclear inner membrane [GO:0005637]; nuclear membrane [GO:0031965]	cytoskeleton-nuclear membrane anchor activity [GO:0140444]; identical protein binding [GO:0042802]; lamin binding [GO:0005521]; microtubule binding [GO:0008017]; protein-membrane adaptor activity [GO:0043495]
SON	microtubule cytoskeleton organization [GO:0000226]; mitotic cytokinesis [GO:0000281]; mRNA processing [GO:0006397]; negative regulation of apoptotic process [GO:0043066]; regulation of cell cycle [GO:0051726]; regulation of mRNA splicing, via spliceosome [GO:0048024]; regulation of RNA splicing [GO:0043484]; RNA splicing [GO:0008380]	nuclear speck [GO:0016607]	DNA binding [GO:0003677]; RNA binding [GO:0003723]
CTPS1	'de novo' CTP biosynthetic process [GO:0044210]; glutamine metabolic process [GO:0006541]; pyrimidine nucleobase biosynthetic process [GO:0019856]	cytoophidium [GO:0097268]; cytoplasm [GO:0005737]	ATP binding [GO:0005524]; CTP synthase activity [GO:0003883]; identical protein binding [GO:0042802]
BCLAF1	apoptotic process [GO:0006915]; cellular response to leukemia inhibitory factor [GO:1990830]; DNA damage response [GO:0006974]; negative regulation of DNA-templated transcription [GO:0045892]; positive regulation of apoptotic process [GO:0043065]; positive regulation of DNA-templated transcription initiation [GO:2000144]; positive regulation of intrinsic apoptotic signaling pathway [GO:2001244]; positive regulation of transcription by RNA polymerase II [GO:0045944]	cytoplasm [GO:0005737]; mediator complex [GO:0016592]; nuclear speck [GO:0016607]; nucleoplasm [GO:0005654]; nucleus [GO:0005634]	DNA binding [GO:0003677]; RNA binding [GO:0003723]; transcription coregulator activity [GO:0003712]
SVIL	actin filament severing [GO:0051014]; actin polymerization or depolymerization [GO:0008154];	actin cytoskeleton [GO:0015629]; cytosol [GO:0005829]; plasma membrane [GO:0005886]	actin filament binding [GO:0051015]; phosphatidylinositol-4,5-

	barbed-end actin filament capping [GO:0051016]		bisphosphate binding [GO:0005546]
PML	apoptotic process [GO:0006915]; branching involved in mammary gland duct morphogenesis [GO:0060444]; cell fate commitment [GO:0045165]; cellular response to interleukin-4 [GO:0071353]; cellular response to leukemia inhibitory factor [GO:1990830]; cellular senescence [GO:0090398]; chromatin remodeling [GO:0006338]; circadian regulation of gene expression [GO:0032922]; DNA damage response, signal transduction by p53 class mediator resulting in cell cycle arrest [GO:0006977]; endoplasmic reticulum calcium ion homeostasis [GO:0032469]; entrainment of circadian clock by photoperiod [GO:0043153]; extrinsic apoptotic signaling pathway [GO:0097191]; fibroblast migration [GO:0010761]; innate immune response [GO:0045087]; intrinsic apoptotic signaling pathway in response to DNA damage [GO:0008630]; intrinsic apoptotic signaling pathway in response to DNA damage by p53 class mediator [GO:0042771]; intrinsic apoptotic signaling pathway in response to endoplasmic reticulum stress [GO:0070059]; intrinsic apoptotic signaling pathway in response to oxidative stress [GO:0008631]; maintenance of protein location in nucleus [GO:0051457]; myeloid cell differentiation [GO:0030099]; negative regulation of angiogenesis [GO:0016525]; negative regulation of cell growth [GO:0030308]; negative regulation of cell population proliferation [GO:0008285]; negative regulation of DNA-templated transcription [GO:0045892]; negative regulation of interleukin-1 beta production [GO:0032691]; negative regulation of mitotic cell cycle [GO:0045930]; negative regulation of telomerase activity [GO:0051974]; negative regulation of telomere maintenance	chromosome, telomeric region [GO:0000781]; cytoplasm [GO:0005737]; cytosol [GO:0005829]; early endosome membrane [GO:0031901]; endoplasmic reticulum membrane [GO:0005789]; nuclear matrix [GO:0016363]; nuclear membrane [GO:0031965]; nucleolus [GO:0005730]; nucleoplasm [GO:0005654]; nucleus [GO:0005634]; PML body [GO:0016605]	cobalt ion binding [GO:0050897]; DNA binding [GO:0003677]; identical protein binding [GO:0042802]; molecular adaptor activity [GO:0060090]; protein heterodimerization activity [GO:0046982]; protein homodimerization activity [GO:0042803]; SMAD binding [GO:0046332]; SUMO binding [GO:0032183]; SUMO transferase activity [GO:0019789]; sumo-dependent protein binding [GO:0140037]; transcription coactivator activity [GO:0003713]; ubiquitin protein ligase activity [GO:0061630]; ubiquitin protein ligase binding [GO:0031625]; ubiquitin-like protein ligase activity [GO:0061659]; zinc ion binding [GO:0008270]

	via telomerase [GO:0032211]; negative regulation of translation in response to oxidative stress [GO:0032938]; negative regulation of ubiquitin-dependent protein catabolic process [GO:2000059]; oncogene-induced cell senescence [GO:0090402]; PML body organization [GO:0030578]; positive regulation of apoptotic process involved in mammary gland involution [GO:0060058]; positive regulation of defense response to virus by host [GO:0002230]; positive regulation of extrinsic apoptotic signaling pathway [GO:2001238]; positive regulation of fibroblast proliferation [GO:0048146]; positive regulation of peptidyl-lysine acetylation [GO:2000758]; positive regulation of protein localization to chromosome, telomeric region [GO:1904816]; positive regulation of signal transduction by p53 class mediator [GO:1901798]; positive regulation of telomere maintenance [GO:0032206]; proteasome-mediated ubiquitin-dependent protein catabolic process [GO:0043161]; protein import into nucleus [GO:0006606]; protein monoubiquitination [GO:0006513]; protein stabilization [GO:0050821]; protein sumoylation [GO:0016925]; protein targeting [GO:0006605]; protein-containing complex assembly [GO:0065003]; protein-containing complex localization [GO:0031503]; regulation of calcium ion transport into cytosol [GO:0010522]; regulation of cell adhesion [GO:0030155]; regulation of cell cycle [GO:0051726]; regulation of circadian rhythm [GO:0042752]; regulation of DNA-templated transcription [GO:0006355]; regulation of double-strand break repair [GO:2000779]; response to cytokine [GO:0034097]; response to gamma radiation [GO:0010332]; response to hypoxia [GO:0001666]; response to UV [GO:0009411]; retinoic acid receptor		

	signaling pathway [GO:0048384]; SMAD protein signal transduction [GO:0060395]; suppression of viral release by host [GO:0044790]; transforming growth factor beta receptor signaling pathway [GO:0007179]		
PDIA4	protein folding [GO:0006457]; response to endoplasmic reticulum stress [GO:0034976]	cell surface [GO:0009986]; endoplasmic reticulum lumen [GO:0005788]; melanosome [GO:0042470]	protein disulfide isomerase activity [GO:0003756]
TMEM43	innate immune response [GO:0045087]; lipid metabolic process [GO:0006629]; nuclear membrane organization [GO:0071763]	endoplasmic reticulum lumen [GO:0005788]; endoplasmic reticulum membrane [GO:0005789]; Golgi apparatus [GO:0005794]; nuclear envelope [GO:0005635]; nuclear inner membrane [GO:0005637]; plasma membrane [GO:0005886]	
C17orf85	7-methylguanosine mRNA capping [GO:0006370]; defense response to virus [GO:0051607]; mRNA export from nucleus [GO:0006406]; mRNA transcription by RNA polymerase II [GO:0042789]; regulatory ncRNA-mediated post-transcriptional gene silencing [GO:0035194]; snRNA export from nucleus [GO:0006408]	cytoplasm [GO:0005737]; nuclear cap binding complex [GO:0005846]; nuclear speck [GO:0016607]; nucleus [GO:0005634]; RNA cap binding complex [GO:0034518]	mRNA binding [GO:0003729]; RNA 7-methylguanosine cap binding [GO:0000340]; RNA binding [GO:0003723]; RNA cap binding [GO:0000339]
THRAP3	positive regulation of transcription by RNA polymerase II [GO:0045944]	mediator complex [GO:0016592]; nuclear speck [GO:0016607]	DNA binding [GO:0003677]; transcription coregulator activity [GO:0003712]
ACTN1	actin cytoskeleton organization [GO:0030036]; actin filament bundle assembly [GO:0051017]; actin filament network formation [GO:0051639]; actin filament organization [GO:0007015]; focal adhesion assembly [GO:0048041]; muscle cell development [GO:0055001]; platelet formation [GO:0030220]; platelet morphogenesis [GO:0036344]; regulation of apoptotic process [GO:0042981]	brush border [GO:0005903]; cell junction [GO:0030054]; cell projection [GO:0042995]; cell-cell junction [GO:0005911]; cortical actin cytoskeleton [GO:0030864]; cytoplasm [GO:0005737]; cytosol [GO:0005829]; extracellular exosome [GO:0070062]; extracellular region [GO:0005576]; extracellular space [GO:0005615]; fascia adherens [GO:0005916]; focal adhesion [GO:0005925]; glutamatergic synapse [GO:0098978]; plasma membrane [GO:0005886]; platelet alpha granule lumen [GO:0031093]; pseudopodium [GO:0031143]; ruffle [GO:0001726]; stress fiber [GO:0001725]; Z disc [GO:0030018]	actin filament binding [GO:0051015]; calcium ion binding [GO:0005509]; double-stranded RNA binding [GO:0003725]; integrin binding [GO:0005178]; nuclear receptor coactivator activity [GO:0030374]; protein homodimerization activity [GO:0042803]; structural constituent of postsynapse [GO:0099186]; transmembrane transporter binding [GO:0044325]; vinculin binding [GO:0017166]
LIMA1	actin filament bundle assembly [GO:0051017]; cell migration [GO:0016477]; cholesterol	actin cytoskeleton [GO:0015629]; brush border membrane [GO:0031526]; cleavage furrow	actin filament binding [GO:0051015]; actin monomer binding [GO:0003785]; cadherin

	homeostasis [GO:0042632]; cholesterol metabolic process [GO:0008203]; intestinal cholesterol absorption [GO:0030299]; negative regulation of actin filament depolymerization [GO:0030835]; ruffle organization [GO:0031529]	[GO:0032154]; cytosol [GO:0005829]; focal adhesion [GO:0005925]; plasma membrane [GO:0005886]; ruffle [GO:0001726]; stress fiber [GO:0001725]	binding [GO:0045296]; metal ion binding [GO:0046872]
KIF14	activation of protein kinase activity [GO:0032147]; cell division [GO:0051301]; cell proliferation in forebrain [GO:0021846]; cerebellar cortex development [GO:0021695]; cerebellar granular layer structural organization [GO:0021685]; cerebellar Purkinje cell layer structural organization [GO:0021693]; cerebral cortex development [GO:0021987]; establishment of protein localization [GO:0045184]; hippocampus development [GO:0021766]; microtubule-based movement [GO:0007018]; mitotic metaphase chromosome alignment [GO:0007080]; negative regulation of apoptotic process [GO:0043066]; negative regulation of integrin activation [GO:0033624]; negative regulation of neuron apoptotic process [GO:0043524]; olfactory bulb development [GO:0021772]; positive regulation of cell population proliferation [GO:0008284]; positive regulation of cytokinesis [GO:0032467]; proteasome-mediated ubiquitin-dependent protein catabolic process [GO:0043161]; regulation of cell adhesion [GO:0030155]; regulation of cell growth [GO:0001558]; regulation of cell maturation [GO:1903429]; regulation of cell migration [GO:0030334]; regulation of G1/S transition of mitotic cell cycle [GO:2000045]; regulation of G2/M transition of mitotic cell cycle [GO:0010389]; regulation of myelination [GO:0031641]; regulation of neuron apoptotic process [GO:0043523]; regulation of Rap protein signal transduction [GO:0032487]; SCF-dependent proteasomal ubiquitin-dependent	cytosol [GO:0005829]; Flemming body [GO:0090543]; kinesin complex [GO:0005871]; membrane [GO:0016020]; microtubule [GO:0005874]; midbody [GO:0030496]; nucleus [GO:0005634]; spindle midzone [GO:0051233]	ATP binding [GO:0005524]; ATP hydrolysis activity [GO:0016887]; microtubule binding [GO:0008017]; microtubule motor activity [GO:0003777]; PDZ domain binding [GO:0030165]; plus-end-directed microtubule motor activity [GO:0008574]; protein kinase binding [GO:0019901]; tubulin binding [GO:0015631]

	protein catabolic process [GO:0031146]; substrate adhesion-dependent cell spreading [GO:0034446]		
SPTAN1	actin cytoskeleton organization [GO:0030036]; actin filament capping [GO:0051693]	cell junction [GO:0030054]; cell projection [GO:0042995]; cortical actin cytoskeleton [GO:0030864]; cytosol [GO:0005829]; extracellular exosome [GO:0070062]; extracellular region [GO:0005576]; extracellular vesicle [GO:1903561]; intracellular membrane-bounded organelle [GO:0043231]; membrane [GO:0016020]; microtubule cytoskeleton [GO:0015630]; plasma membrane [GO:0005886]; specific granule lumen [GO:0035580]; spectrin [GO:0008091]; tertiary granule lumen [GO:1904724]	actin binding [GO:0003779]; actin filament binding [GO:0051015]; cadherin binding [GO:0045296]; calcium ion binding [GO:0005509]; calmodulin binding [GO:0005516]; structural constituent of cytoskeleton [GO:0005200]
FLNA	actin crosslink formation [GO:0051764]; actin cytoskeleton organization [GO:0030036]; adenylate cyclase-inhibiting dopamine receptor signaling pathway [GO:0007195]; angiogenesis [GO:0001525]; blood coagulation, intrinsic pathway [GO:0007597]; blood vessel remodeling [GO:0001974]; cell-cell junction organization [GO:0045216]; cerebral cortex development [GO:0021987]; cilium assembly [GO:0060271]; cytoplasmic sequestering of protein [GO:0051220]; early endosome to late endosome transport [GO:0045022]; epithelial to mesenchymal transition [GO:0001837]; establishment of protein localization [GO:0045184]; establishment of Sertoli cell barrier [GO:0097368]; formation of radial glial scaffolds [GO:0021943]; heart morphogenesis [GO:0003007]; megakaryocyte development [GO:0035855]; mitotic spindle assembly [GO:0090307]; mRNA transcription by RNA polymerase II [GO:0042789]; negative regulation of apoptotic process [GO:0043066]; negative regulation of DNA-binding transcription factor activity [GO:0043433]; negative regulation	actin cytoskeleton [GO:0015629]; actin filament [GO:0005884]; actin filament bundle [GO:0032432]; apical dendrite [GO:0097440]; axonal growth cone [GO:0044295]; brush border [GO:0005903]; cell-cell junction [GO:0005911]; cortical cytoskeleton [GO:0030863]; cytoplasm [GO:0005737]; cytosol [GO:0005829]; dendritic shaft [GO:0043198]; extracellular exosome [GO:0070062]; extracellular region [GO:0005576]; focal adhesion [GO:0005925]; glutamatergic synapse [GO:0098978]; glycoprotein Ib-IX-V complex [GO:1990779]; membrane [GO:0016020]; Myb complex [GO:0031523]; nucleolus [GO:0005730]; nucleus [GO:0005634]; perikaryon [GO:0043204]; perinuclear region of cytoplasm [GO:0048471]; plasma membrane [GO:0005886]; postsynapse [GO:0098794]; trans-Golgi network [GO:0005802]; Z disc [GO:0030018]	actin filament binding [GO:0051015]; cadherin binding [GO:0045296]; DNA-binding transcription factor binding [GO:0140297]; Fc-gamma receptor I complex binding [GO:0034988]; G protein-coupled receptor binding [GO:0001664]; GTPase binding [GO:0051020]; kinase binding [GO:0019900]; potassium channel regulator activity [GO:0015459]; protein homodimerization activity [GO:0042803]; protein kinase C binding [GO:0005080]; RNA binding [GO:0003723]; SMAD binding [GO:0046332]; small GTPase binding [GO:0031267]; transmembrane transporter binding [GO:044325]

	of neuron projection development [GO:0010977]; negative regulation of protein catabolic process [GO:0042177]; negative regulation of transcription by RNA polymerase I [GO:0016479]; platelet aggregation [GO:0070527]; positive regulation of actin filament bundle assembly [GO:0032233]; positive regulation of axon regeneration [GO:0048680]; positive regulation of canonical NF-kappaB signal transduction [GO:0043123]; positive regulation of integrin-mediated signaling pathway [GO:2001046]; positive regulation of neural precursor cell proliferation [GO:2000179]; positive regulation of neuron migration [GO:2001224]; positive regulation of platelet activation [GO:0010572]; positive regulation of potassium ion transmembrane transport [GO:1901381]; positive regulation of protein import into nucleus [GO:0042307]; positive regulation of substrate adhesion-dependent cell spreading [GO:1900026]; protein localization to bicellular tight junction [GO:1902396]; protein localization to cell surface [GO:0034394]; protein localization to plasma membrane [GO:0072659]; protein stabilization [GO:0050821]; receptor clustering [GO:0043113]; regulation of cell migration [GO:0030334]; regulation of membrane repolarization during atrial cardiac muscle cell action potential [GO:1905000]; regulation of membrane repolarization during cardiac muscle cell action potential [GO:1905031]; release of sequestered calcium ion into cytosol [GO:0051209]; semaphorin-plexin signaling pathway [GO:0071526]; synapse organization [GO:0050808]; tubulin deacetylation [GO:0090042]; wound healing, spreading of cells [GO:0044319]		

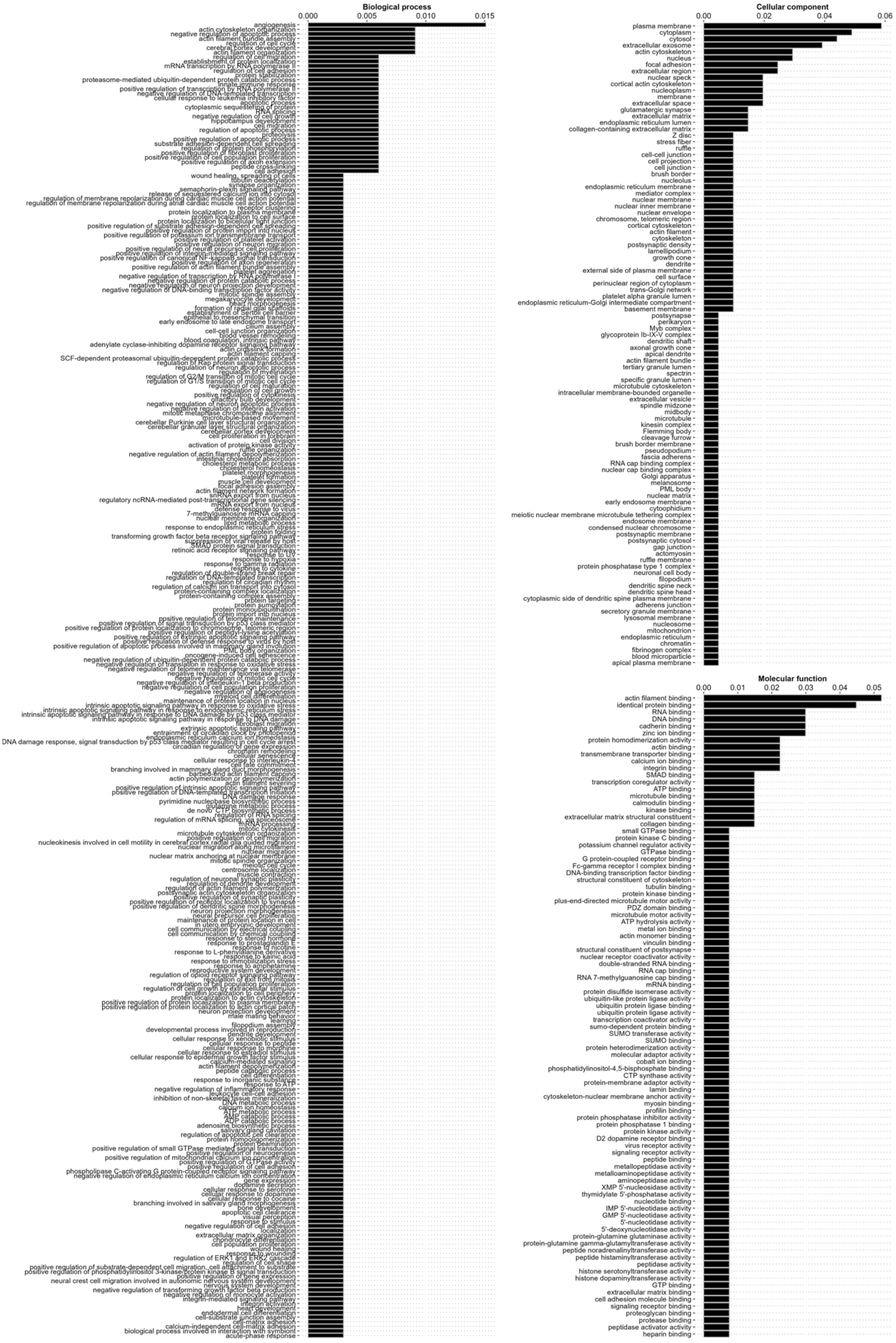

Supplementary Figure 3. EMT increases the binding to intermediate filaments of proteins that regulate the cell-extracellular matrix adhesion, cytoskeleton, cell shape and cell motility, while ALD-R491 reduces binding of RNA metabolism and function. The full list of GO annotations of the vimentin interactome upon treatment with TGF-β1 (A) or additional ALD-R491 (B), shown as

percentages, with the Biological function (left), Cellular compartment (top right), and Molecular activity (bottom right panels), as indicated.

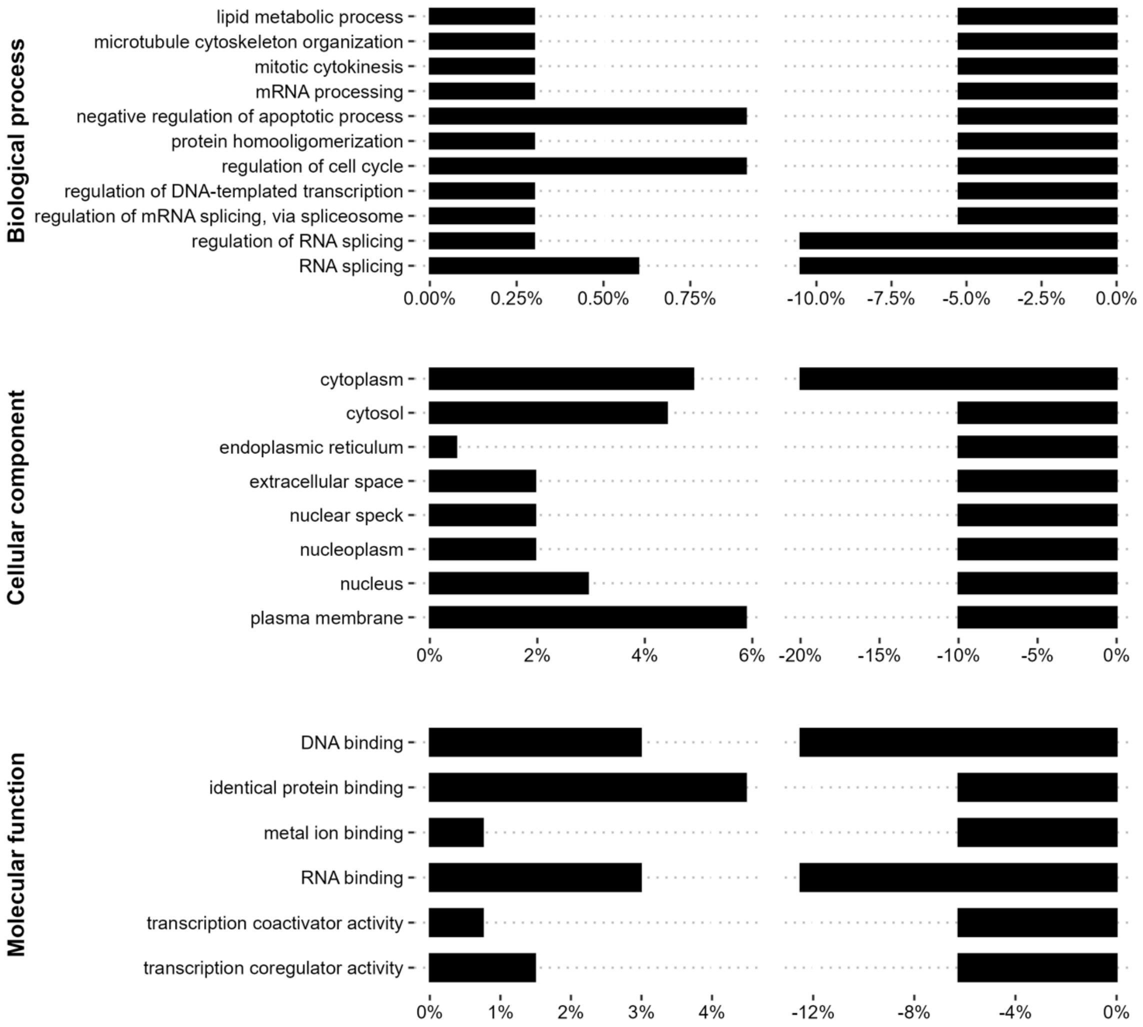

Supplementary Figure 4. EMT increases, while ALD-R491 decreases, the binding of proteins that regulate RNA metabolism and function to intermediate filaments. GO annotations of changes of vimentin interactome of lung cancer cells upon treatment with TGF-β1 (left), or between TGF-β1 only and TGF-β1 with additional ALD-R491 (right panel), shown as percentages, with the Biological function (top), Cellular compartment (middle), and Molecular activity (lower panels), as indicated.

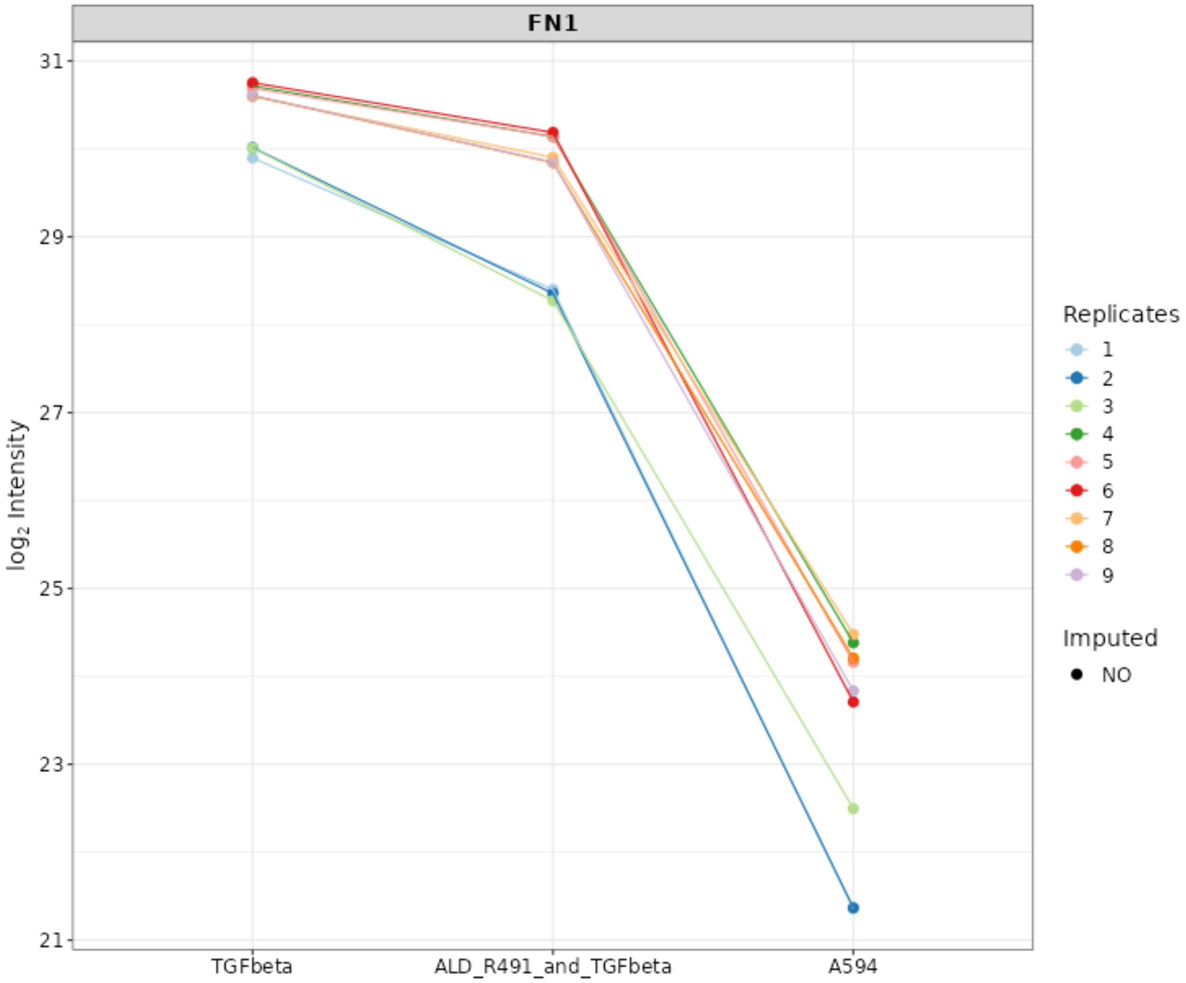

Supplementary Figure 5. Example of Mass Spectrometry-based quantitative proteomic data. Each of the three, separate, biological replicates were divided and analysed in three technical replicates. The nine samples were analysed, and peptides identified, as outlined in the material and methods section. The examples show the levels of fibronectin 1 peptides identified in the analysis, in the nine samples, as indicated. Black dot indicates imputed value (absent in this example).

Supplementary Table 3. The Gene ontology for EMT-induced vimentin binding proteins which were changed upon treatment with ALD-R491, with regards to Biological process, Cellular component and Molecular function, as indicated.

Protein	Biological process	Cellular component	Molecular function
SON	microtubule cytoskeleton organization [GO:0000226]; mitotic cytokinesis [GO:0000281]; mRNA processing [GO:0006397]; negative regulation of apoptotic process [GO:0043066]; regulation of cell cycle [GO:0051726]; regulation of mRNA splicing, via spliceosome [GO:0048024]; regulation of RNA splicing [GO:0043484]; RNA splicing [GO:0008380]	nuclear speck [GO:0016607]	DNA binding [GO:0003677]; RNA binding [GO:0003723]
ALDH3A1	cellular aldehyde metabolic process [GO:0006081]; lipid metabolic process [GO:0006629]; xenobiotic metabolic process [GO:0006805]	cytoplasm [GO:0005737]; cytosol [GO:0005829]; endoplasmic reticulum [GO:0005783]; extracellular space [GO:0005615]; plasma membrane [GO:0005886]	3-chloroallyl aldehyde dehydrogenase activity [GO:0004028]; alcohol dehydrogenase (NADP+) activity [GO:0008106]; aldehyde dehydrogenase (NAD+) activity [GO:0004029]; aldehyde dehydrogenase [NAD(P)+] activity [GO:0004030]; benzaldehyde dehydrogenase (NAD+) activity [GO:0018479]
FUS	amyloid fibril formation [GO:1990000]; mRNA stabilization [GO:0048255]; positive regulation of double-strand break repair via homologous recombination [GO:1905168]; protein homooligomerization [GO:0051260]; regulation of DNA-templated transcription [GO:0006355]; regulation of RNA splicing [GO:0043484]; regulation of transcription by RNA polymerase II [GO:0006357]; RNA splicing [GO:0008380]	cytoplasm [GO:0005737]; intracellular non-membrane-bounded organelle [GO:0043232]; nucleoplasm [GO:0005654]; nucleus [GO:0005634]	chromatin binding [GO:0003682]; DNA binding [GO:0003677]; identical protein binding [GO:0042802]; metal ion binding [GO:0046872]; molecular condensate scaffold activity [GO:0140693]; mRNA 3'-UTR binding [GO:0003730]; RNA binding [GO:0003723]; transcription coactivator activity [GO:0003713]; transcription coregulator activity [GO:0003712]

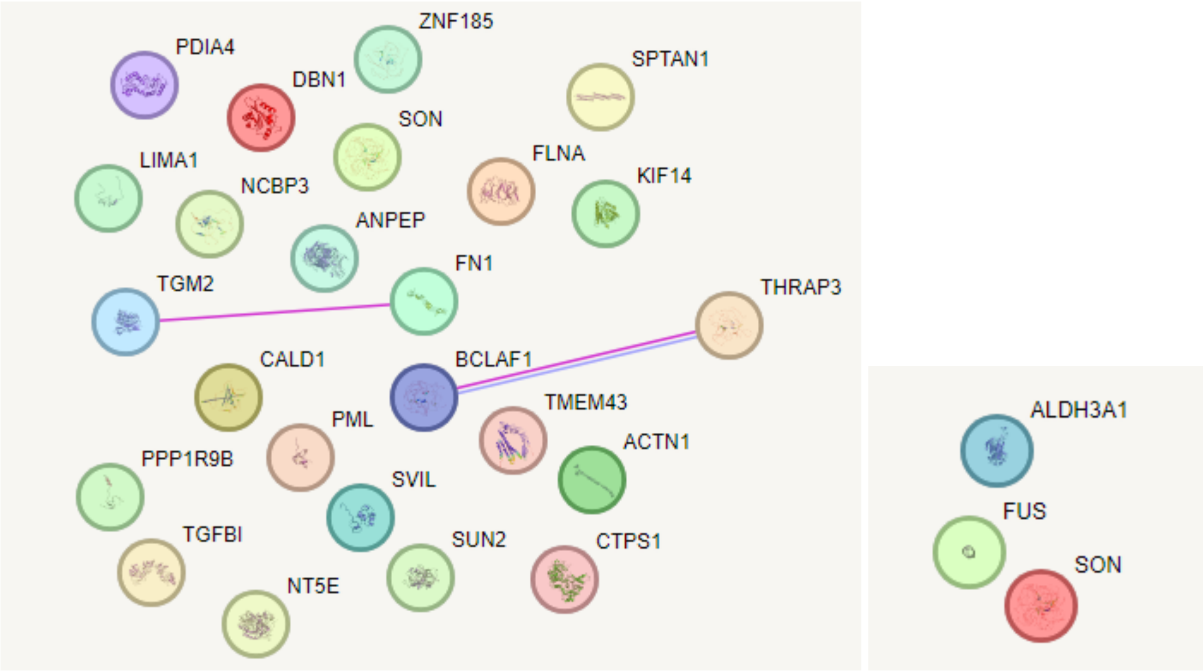

Supplementary Figure 6. STRING analysis of protein-to-protein interactions between proteins of the vimentin interactome. The changes of the vimentin interactome upon TGF-β1-induced EMT (left), and upon additional treatment with ALD-R491 (right)

.
